# Orthology inference at scale with FastOMA

**DOI:** 10.1038/s41592-024-02552-8

**Published:** 2025-01-03

**Authors:** Sina Majidian, Yannis Nevers, Ali Yazdizadeh Kharrazi, Alex Warwick Vesztrocy, Stefano Pascarelli, David Moi, Natasha Glover, Adrian M. Altenhoff, Christophe Dessimoz

**Affiliations:** 1 Department of Computational Biology, University of Lausanne, Lausanne, Switzerland; 2 Swiss Institute of Bioinformatics, Lausanne, Switzerland; 3 Department of Computer Science, ETH Zurich, Zurich, Switzerland

**Keywords:** Software, Genome informatics, Evolution, Evolutionary biology, Comparative genomics

## Abstract

The surge in genome data, with ongoing efforts aiming to sequence 1.5 M eukaryotes in a decade, could revolutionize genomics, revealing the origins, evolution and genetic innovations of biological processes. Yet, traditional genomics methods scale poorly with such large datasets. Here, addressing this, ‘FastOMA’ provides linear scalability for orthology inference, enabling the processing of thousands of eukaryotic genomes within a day. FastOMA maintains the high accuracy and resolution of the well-established Orthologous Matrix (OMA) approach in benchmarks. FastOMA is available via GitHub at https://github.com/DessimozLab/FastOMA/.

## Abstract (web summary)

FastOMA achieves fast and accurate orthology inference, with linear scalability.

## Main

Within the decade, the Earth BioGenome initiative aims to sequence 1.5 M eukaryotes^1^. This paves the way for understanding how all species evolved from life’s common origin. Yet, due to processing limitations, even the thousands of genomes we have access to today are studied only piecemeal in practice. A fundamental step to comparative genomics analyses is to identify orthologs, genes of common ancestry that originated by speciation events^2^. When performed systematically, orthology delineation conveys how sequences were gained, lost or duplicated, assuming that their basic mode of inheritance is vertical descent. Deriving orthology enables many types of downstream analysis, such as annotation propagation, phylogenomics or phylogenetic profiling^3^.

State-of-the-art orthology methods face acute scalability issues^4^. Methods relying on all-against-all sequence comparisons can no longer keep up with today’s data, let alone tomorrow’s. For state-of-the-art pipelines such as our own Orthologous MAtrix (OMA) algorithm and database^5,6^, this translates to >10 million central processing unit (CPU) hours to derive the orthology relationships of >2000 genomes that have been processed thus far. Methods relying on whole-genome alignment, such as TOGA (Tool to infer Orthologs from Genome Alignments)^7^, are more efficient, but the genome alignment requirement limits their applicability to relatively closely related species. While small-scale comparative genomics has achieved remarkable progress, a more integrated, large-scale approach would be transformative.

To address this challenge, we introduce FastOMA, which dramatically speeds up orthology inference without sacrificing accuracy or resolution.

**Fig. 1:**
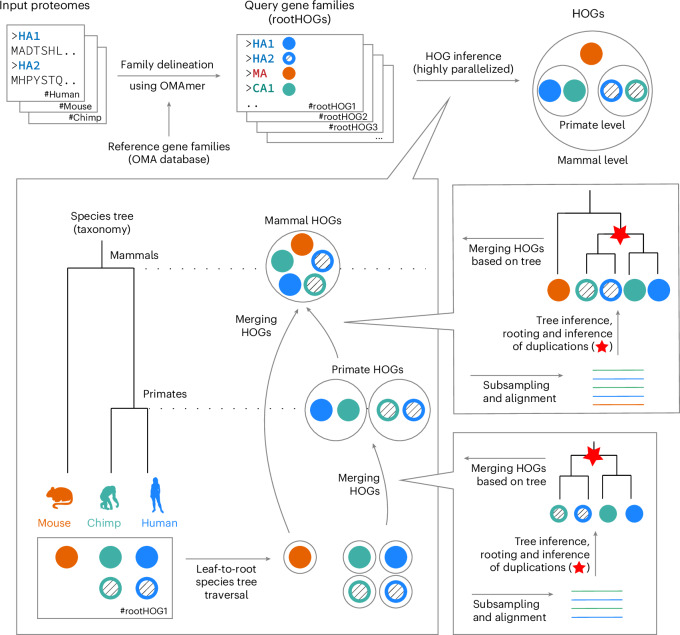
FastOMA algorithm overview. Input proteomes are mapped to reference gene families using the OMAmer software, forming hierarchical orthologous groups (HOGs) at the root level (rootHOGs), see Methods. HOGs are inferred using a ‘bottom-up’ approach, starting from the leaves of the species tree and moving towards the root. At each taxonomic level, HOGs from the child level are merged, resulting in HOGs at the current level. To decide which HOGs should be merged, sequences from the child HOGs are used to create a MSA^18^, followed by gene tree inference^19^ to identify speciation and duplication events^20^. Child HOGs are merged if their genes evolved through speciation (see Methods and Supplementary Information 1 for details). Credit: human silhouette, T. Michael Keesey (Public Domain Mark 1.0); chimpanzee silhouette, Jonathan Lawley (CC0 1.0 Universal); mouse silhouette, Soledad Miranda-Rottman (CC BY 3.0), PhyloPic.

FastOMA is a complete rewrite of the OMA algorithm focused on scalability from the ground up (Fig. 1). By combining ultrafast homology clustering using *k*-mers, taxonomy-guided subsampling and a highly efficient parallel computing approach, it achieves linear performance in the number of input genomes. First, we leverage our current knowledge of the sequence universe (with its evolutionary information stored in the OMA database) to efficiently place new sequences into coarse-grained families (hierarchical orthologous groups (HOGs) at the root level) using the alignment-free *k*-mer-based OMAmer tool^8^. In an attempt to detect homology among unplaced sequences (which could belong to families that are absent from our reference database), we then perform a round of clustering using the highly scalable Linclust software^9^. Next, we resolve the nested structure of the HOGs (Supplementary Information 1) corresponding to each ancestor, in an efficient leaf-to-root traversal of the species tree. By avoiding sequence comparisons across different families, the number of computations is drastically reduced compared with conventional approaches (see Methods for details).

**Fig. 2:**
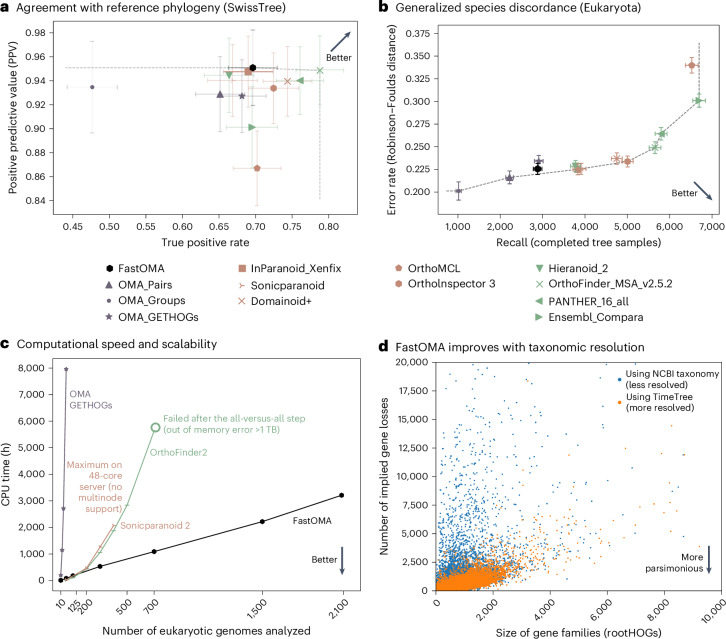
FastOMA is not only fast but also accurate. **a**, QfO benchmark^10^, agreement with SwissTree reference phylogeny covering 19 manually curated gene trees. The error bars indicate 95% confidence intervals comparing FastOMA with EnsemblCompara^21^, Domainoid^22^, OrthoMCL^23^, Ortholnspector^24^, sonicparanoid, PANTHER^25^, OrthoFinder, Hieranoid^26^ and the OMA family including OMA pairs, OMA groups and OMA GETHOGs (graph-based efficient technique for HOGs)^27–29^. **b**, QfO benchmarking of the generalized species discordance test on the Eukaryota clade, where the gene tree inferred from orthologous genes is compared with the reference species tree considering up to 3,000 gene trees per method (see Supplementary Information 2.1 for details). **c**, A computation time comparison of FastOMA and state-of-the-art alternatives. **d**, The impact of species tree resolution on the complexity of the gene family evolutionary scenario (proxied by the number of gene losses over the gene family history). Each point represents a gene family (a rootHOG), whereby the size of a gene family corresponds to the number of genes in it^30^ (the figure is truncated to focus on the most relevant region; see Supplementary Fig. 24 for a version with all data, and see Methods for the implied losses calculation).

FastOMA has high scalability without sacrificing accuracy in a diverse range of benchmarks. We assessed the accuracy of FastOMA on the Quest for Orthologs (QfO) suite of benchmarks^10^. FastOMA retains OMA’s high precision accuracy and even improves upon it in terms of recall, positioning it on the Pareto frontier of orthology inference methods. For instance, on the SwissTree reference gene phylogeny benchmark, FastOMA outperforms other methods with a precision of 0.955 in reference gene phylogenies (Fig. 2a). With a recall in line with most state-of-the-art methods (0.69, lower than those of Panther and OrthoFinder), the balance of these metrics indicates a well-tuned approach to orthology inference, with a focus on minimizing false positives. Likewise, on the generalized species tree benchmark at the Eukaryota level, FastOMA is among those with the lowest topological error, with a normalized Robinson–Foulds distance—the number of different edges between two trees normalized by the total number of internal edges—of 0.225 to the reference tree, at moderate recall (Fig. 2b and Supplementary Information 2–17).

A key achievement of FastOMA is its linear scaling behavior (Fig. 2c), which opens up the possibility of processing extensive datasets rapidly. FastOMA inferred orthology among all 2,086 eukaryotic UniProt reference proteomes in under 24 h, using 300 CPU cores. In the same timespan, the original OMA algorithm could process only 50 genomes. Even methods optimized for speed such as OrthoFinder^11^ or SonicParanoid^12^ still exhibit quadratic time complexity (Fig. 2c). Thus, FastOMA’s linear scalability breaks new ground.

The initial sequence placement step using OMAmer helps FastOMA achieve its speed, but the subsequent alignment and tree inference steps are critical for its accuracy. Indeed, sequence placement alone is considerably less accurate than state-of-the-art methods in benchmarks (Supplementary Information 3).

FastOMA exploits known taxonomic relationships to reduce the number of sequence comparisons. By default, it relies on the commonly used National Center for Biotechnology Information (NCBI) taxonomy^13,14^, but users can specify any reference species phylogeny as input. To assess the impact of the resolution of the input tree on orthology accuracy, we compared FastOMA’s performance on UniProt reference proteomes with a more resolved species tree derived from the TimeTree resource^15^. Compared with the NCBI taxonomy, this resulted in improved ortholog predictions, with more parsimonious gene family evolution history, lowering the number of implied gene losses across all gene families (Fig. 2d). FastOMA is also robust to errors artificially introduced in the species taxonomy (Supplementary Figs. 18–20). FastOMA can thus use advances in taxonomic knowledge for better orthology predictions and will benefit from the higher resolution that is brought by new genomic sequences from large-scale sequencing projects.

FastOMA contains additional features that make it easier to deal with complex and noisy genomic data. It is designed to handle multiple isoforms for the genes resulting from alternative splicing and select the most evolutionarily conserved ones, and can also deal with fragmented gene models^16^. Both features lead to noticeable improvements in FastOMA inferences (Supplementary Information 9 and 10). As it uses the same data structure as OMA, FastOMA benefits from its rich ecosystem of downstream applications, including phylogenetic profiling, efficient gene family visualization, ancestral synteny inference and advanced phylostratigraphy, enabling researchers to trace gene family histories and understand gene emergence, duplication and loss events^5,17^.

In conclusion, the FastOMA algorithm offers a unique solution for accurate orthology inference, making it possible to study evolutionary history at the scale of massive genomics projects. Future work will aim to further refine orthology inference by integrating structural protein data to improve resolution at deeper evolutionary levels, as well as gene order conservation as an additional layer of information.

## Methods

### FastOMA algorithm outline

FastOMA is a method for inferring orthology relationships. The input to FastOMA includes the proteome sets of species and the species tree. The FastOMA algorithm consists of two main steps: finding rootHOGs and inferring the nested structure of HOGs (Fig. 1).

### Step 1: FastOMA gene family inference

The FastOMA algorithm infers gene families from the provided proteomes. The process begins by mapping the input proteomes onto the reference HOGs (Supplementary Information 1) using the OMAmer tool (Fig. 1). Proteins mapped to the same reference HOG are then grouped together, forming query rootHOGs, with the exclusion of proteins already present in the database. Thus, proteins in the database reference HOGs are not used in the next steps in FastOMA.

Although each rootHOG ideally represents a single gene family, instances may arise where a gene family of query proteomes is split among multiple rootHOGs. To address this, FastOMA tries to find those query rootHOGs that are associated with the same gene family. FastOMA leverages the ability of OMAmer to report multiple rootHOGs to which the sequences could be mapped, along with their score. This score (‘family_p’) is the *P* value of having as many or more *k*-mers in common between the protein sequence and the HOG under a binomial distribution, reported in negative natural logarithm. Considering a minimum threshold of 70 (by default), we construct a graph of rootHOGs, where each node represents a query rootHOG. In such a graph, we add an edge between two nodes (rootHOGs) when a minimum of ten proteins (by default) are mapped to both query rootHOGs and it represents at least either 80% of all proteins mapping to the bigger rootHOG or 90% of those mapping to the smallest one. This ensures a high overlap of protein content of the merged rootHOG. Finally, we group the members of all HOGs in each highly connected component of this graph in a single query rootHOG.

It is worth noting that some proteins may not be assigned to any reference HOGs owing to no recognizable homologs in the reference database. In addition, there is a scenario where only one protein is mapped to the rootHOG, referred to as a singleton, representing an individual rather than a group^5^. To ensure those genes are not lost to FastOMA’s orthology inference, these singletons and unmapped sequences are combined into a FASTA file on which we run Linclust, the clustering tool from the MMseqs package^9^. This yields new query rootHOGs.

Critically, assigning proteins to rootHOGs (gene families) allows us to avoid unnecessary all-against-all comparisons of unrelated proteins (those without homology), thanks to the speed of OMAmer and Linclust. All the query rootHOGs are written as FASTA files to be used in the next step and can be handled in parallel.

Notably, the OMA team provides regular updates to the OMA database, increasing the number and diversity of species included in the database used by OMAmer. This results in higher resolution for *k*-mer-based grouping. As more taxa get included, we foresee FastOMA’s inference will improve as more sequences are placed into rootHOGs.

### Step 2: FastOMA orthology inference

For every query rootHOG, FastOMA infers the nested structure of the HOG (as depicted in Fig. 1). The objective is to identify the genes that are grouped together at each taxonomic level as a HOG, which means they descended from a single gene at that specific level. Note that the number of HOGs at each level reflects the number of copies of the gene present in the ancestral species.

To achieve this, FastOMA follows a bottom-up approach by traversing the species tree. Starting from the leaves of the tree (extant species), each gene in the species’ proteome is treated as a HOG. At each level in the traversal, certain HOGs from the child level are combined. The determination of which HOGs will be merged is guided by a gene tree containing the proteins of species descending from this node. The merging is done for all HOGs that descended from the same common ancestor by a speciation event. The entire process is detailed below:

#### Gene tree inference

All the proteins in HOGs at the child level are collectively used for generating a multiple sequence alignment (MSA) using the MAFFT package^18^. As part of the FastOMA Python script, the MSA undergoes column-wise trimming with a default threshold of 0.2, meaning that we remove columns of the MSA that have more than 80% gap elements (Supplementary Information 5). Aligned sequences (rows in MSA) that exceed a default threshold of >50% gaps are subsequently removed. However, we keep them in the HOG, but they are not used for tree inference. Subsequently, we employ FastTree^19^ to infer the gene tree, and this tree is rooted using the midpoint approach.

To expedite the orthology inference process at deeper levels of the trees where the number of children is prohibitively high, we implement a subsampling approach, retaining only a specified number of proteins per HOG (Supplementary Figs. 12–14; by default, 20 proteins are randomly selected) used for the MSA and tree inference. The unsampled sequences will have the same fate as the rest of the proteins in the same group at the defined taxonomic level.

Note that the subsampling strategy is key to the speed of FastOMA, and expectedly, there is a trade-off between accuracy and speed. Our benchmarking results indicate that FastOMA performs well with the subsampling approach, but users can change the degree of the subsampling in the parameter file.

#### Duplication and speciation event labeling

Each internal node in the gene tree is classified as either a duplication or a speciation event using the species overlap method^20^. For each node in the gene tree, this involves calculating the ratio of the number of shared species between its two subtrees divided by the number of all species (union). If the ratio equals zero, the node is labeled as a speciation event; otherwise, it is labeled as a duplication event. When the species overlap ratio is less than 0.1 (as per default settings), indicating very low support for a duplication event, all leaves from the child subtree with the least number of proteins are excluded from merging decisions (described in ‘HOG merging’ section). In other words, these proteins will stay in the corresponding HOGs as in the previous taxonomic level, and only the taxonomic label of the HOG is updated to the current taxonomic level (assuming no other merging happens in another part of the gene tree for this HOG). This is done to ensure that errors in gene annotation or inaccurate tree inference only minimally affect the orthology inference.

#### HOG merging

Starting from the root of the gene tree, evidence of a speciation event (that is, the internal node annotated as a speciation event due to no species overlap) prompts the merging of the HOGs of the leaves descending from the nodes. This is achieved by constructing a HOG graph, where each node represents a HOG. An edge is introduced between HOG1 and HOG2 if protein 1 (located in HOG1) and protein 2 (in HOG2) coalesce at a speciation event in the gene tree. Subsequently, each connected component within this graph constitutes a HOG at the current level of the species tree. Furthermore, FastOMA has a mechanism to handle spuriously merged subHOGs; at the deeper taxonomy level, when genes within a subHOG coalesce at a duplication event in the gene tree, FastOMA splits the subHOG into two, ensuring copies of ancestral genes are not co-present in a subHOG.

#### Inferring orthology relationship

Once the species tree traversal is complete, the nested structure of the query HOG is fully resolved. From the HOG structure inferred this way, all orthology and paralogy relationships can be efficiently deduced.

#### Note on parallelization

Scalability has been a major challenge in the field of orthology inference highlighted by the QfO community for many years^4,10,31^. FastOMA is optimized to process taxonomic levels in parallel (when possible) by inferring HOGs at all taxonomic levels, accounting for dependencies among child HOGs, that is, a node will be processed after all its child nodes are processed. To optimize parallelization efficiency by avoiding unnecessary overheads of Nextflow and Slurm management workflows, FastOMA groups approximately 150 small- to medium-sized query rootHOGs together, treating them as a single job. Conversely, large rootHOGs are processed individually (to infer nested structure of HOGs) for optimal performance using Python-future for which taxonomic parallelization is activated. The default rootHOG file size threshold for this purpose is 400,000 bytes, or ~500 proteins (Supplementary Information 11).

### FastOMA outputs

The main output of FastOMA is an OrthoXML file that stores HOGs and their nested structures, allowing to reconstruct their evolutionary histories. Furthermore, FastOMA reports the protein list in each rootHOG (gene family) in TSV format. A final FastOMA output is a list of proteins in strict orthologous groups, wherein all genes within the group are orthologous to each other, which can be used as marker genes for phylogenetic analyses^32,33^. Besides, the user can store the gene trees and MSAs of the subsampled HOGs for all taxonomic levels.

### Isoform selection

FastOMA is capable of handling proteomes that feature multiple protein isoforms for a gene due to alternative splicing. Users can provide an isoform file where each row lists comma-separated protein IDs associated with a gene. FastOMA selects the isoform with the highest ‘family_p’ score, the one with the best fit to known proteins in the reference rootHOG based on *k*-mer content. For the evaluation of isoform selection, we used the UniProt reference proteomes and their splice information (https://ftp.uniprot.org/pub/databases/uniprot/current_release/knowledgebase/reference_proteomes/Eukaryota).

### FastOMA software

The FastOMA codebase is composed of multiple subpackages written in Python. FastOMA benefits from the Nextflow workflow to parallelize different steps and subpackages considering the dependencies modeled as a direct acyclic graph (Supplementary Information 11). The software is publicly available on GitHub (https://github.com/DessimozLab/FastOMA) and on DockerHub (https://hub.docker.com/r/dessimozlab/fastoma).

### Time comparison on eukaryotic dataset

We considered all 2,181 eukaryotic UniProt reference proteomes (accessed on 25 January 2023) and filtered them to keep those with a minimum BUSCO (benchmarking universal single-copy orthologs) completeness of 50%, resulting in 2,086 proteomes in total. We ran SonicParanoid, OrthoFinder and FastOMA on datasets with different sizes ranging from 10 to 2,086 species. OrthoFinder 2.5.4 was run in two steps. First, to generate all-against-all sequence comparisons, we used the -op parameter to generate and execute command lines for Diamond. Then, the rest of OrthoFinder was conducted. SonicParanoid 2.0.4 was used with default parameters using 48 CPUs with a limit of 3 days wall clock. It is neither possible to parallelize SonicParanoid2 on different computation nodes nor to feed it with the result of Diamond; hence, we could not obtain compute time for the larger datasets during the mentioned time limit. For FastOMA, the NCBI tree was used by downloading via the ETE3 package^14^. The comparison of tools in terms of wall-clock time in hours is reported in Supplementary Fig. 25. The Diamond part of OrthoFinder and all steps of FastOMA use different nodes on the cluster, so the reported wall-clock time might have been affected by the availability of nodes at the time of each run. However, the CPU times reported in Fig. 2c are more accurate.

### Analysis on tree resolution

We ran FastOMA on both the TimeTree and the NCBI tree. For the TimeTree analysis, we uploaded the list of species names to the TimeTree webserver^15^ (https://timetree.org). This resulted in a species tree with 1,757 leaves since some of the species were not available in TimeTree. We ran FastOMA with default parameters on the dataset of 1,757 proteomes and with both the TimeTree tree and NCBI tree as the species tree. We used pyHAM^30^ for calculating the implied gene losses.

To calculate the estimated proportion of proteomes composed of fragments, we ran OMArk^16^ v0.3 on all proteomes. We used the BUSCO statistics downloaded from the UniProt website for the full eukaryotic dataset.

We also conducted another analysis to study the impact of the species tree for the QfO dataset where five pairs of species are swapped. The results are provided in Supplementary Information 7 and Supplementary Figs. 18–20, where FastOMA shows a moderate level of robustness. However, having an erroneous species tree impacted the orthology inference by introducing false positives.

To conclude, we highlight that the orthologous and paralogous genes are found using the species overlap method on the gene tree and the species tree is used to determine the order of comparisons, defining the HOG structure. Thus, a fully resolved species tree is not needed to infer orthology information with FastOMA. However, errors in the species tree can potentially propagate through the orthology inference process.

### Benchmarking against the QfO reference proteome set

We ran FastOMA on the 78 reference proteomes used in the QfO benchmark and the associated standard species trees as input. We then submitted the results to the QfO benchmarking service^4,10,31^^,34^ and obtained the results on the 11 available benchmarks. In these benchmarks, FastOMA is compared with several state-of-the-art methods that are available in the QfO public resource, including EnsemblCompara^21^, Domainoid^22^, OrthoMCL^23^, Ortholnspector^24^, sonicparanoid^35^, PANTHER^25^, OrthoFinder^11^, Hieranoid^26^ and the OMA family^27–29,36^. QfO analysis is described in detail in Supplementary Information 2. The orthogroup benchmarking for the clade Bilateria^37^ is provided in Supplementary Information 8.

### Analysis of the QfO reference proteome set using InterProScan classification of protein families

To study the influence of the OMA database and OMAmer on the performance of FastOMA, we replaced the first part of the procedure, normally done by placing query genes into the OMA database rootHOGs with OMAmer, with InterProScan. We used InterProScan to group the QfO proteomes into gene families predefined by InterProScan^38^. To do so, we first ran InterProScan with the argument -appl Pfam on the QfO dataset, which grouped the proteins into InterProScan families^39^. Then, we created the rootHOG with those groups, maintaining the same InterProScan family identifier. Then, we ran the rest of FastOMA on these rootHOG FASTA files. The QfO benchmarking results are shown in Supplementary Information 6 and Supplementary Figs. 15–17. Note that a user can provide their own initial grouping of proteins to be used with FastOMA. This could be put in practice in two ways: (1) running the last two processes of FastOMA.nf (hog_rest and collect_subhog) on the user’s protein family in FASTA format or (2) providing group mapping of proteins in the OMAmer format.

### Computations

All the analyses were conducted on the high-performance computer cluster of the University of Lausanne that houses 96 computation nodes. Each node is equipped with two 24-core AMD (Advanced Micro Devices) CPUs, totaling 48 cores per node. Data were written and read on a 150 TB SSD (solid-state drive) scratch drive. For the QfO analysis, most steps of FastOMA needed less than 10 GB of memory, with a maximum of 32 GB.

### Reporting summary

Further information on research design is available in the Nature Portfolio Reporting Summary linked to this article.

## Online content

Any methods, additional references, Nature Portfolio reporting summaries, source data, extended data, supplementary information, acknowledgements, peer review information; details of author contributions and competing interests; and statements of data and code availability are available at 10.1038/s41592-024-02552-8.

## Supplementary information

Supplementary Information Supplementary Information 1–11, Table 1 and Figs. 1–25.

Reporting Summary

Peer Review File

## Data Availability

UniProt reference proteomes and splice information (_additional.fasta.gz) were downloaded from https://ftp.uniprot.org/pub/databases/uniprot/current_release/knowledgebase/reference_proteomes/Eukaryota. The 2020 version of QfO proteomes was downloaded from the EBI repository at http://ftp.ebi.ac.uk/pub/databases/reference_proteomes/previous_releases/qfo_release-2020_04_with_updated_UP000008143/. The OMAmer database used in this study is available at https://omabrowser.org/All/LUCA.h5. The OMAmer database, an archive of FastOMA code, the TimeTree with annotation of internal nodes of 1,757 species in Newick format, the UniProt IDs and the inferred HOG for 1,757 eukaryotic species in OrthoXML format are all deposited on Zenodo at 10.5281/zenodo.10403053 (ref. ^40^).

## Code availability

FastOMA is free open-source software (Mozilla Public License 2.0) available via GitHub at https://github.com/DessimozLab/FastOMA. We used the publicly available code for the QfO benchmarking test which is available via GitHub at https://github.com/qfo/benchmark-webservice. A copy of the FastOMA software is available via Zenodo at 10.5281/zenodo.10403053 (ref. ^40^).

## References

[1] Lewin, H. A. et al. Earth BioGenome Project: sequencing life for the future of life. *Proc. Natl Acad. Sci. USA* **115**, 4325–4333 (2018). 29686065 10.1073/pnas.1720115115 PMC5924910

[2] Fitch, W. M. Distinguishing homologous from analogous proteins. *Syst. Zool.* **19**, 99–113 (1970). 5449325

[3] Glover, N. et al. Advances and applications in the Quest for Orthologs. *Mol. Biol. Evol.* **36**, 2157–2164 (2019). 31241141 10.1093/molbev/msz150 PMC6759064

[4] Linard, B. et al. Ten years of collaborative progress in the Quest for Orthologs. *Mol. Biol. Evol*. 10.1093/molbev/msab098 (2021). 10.1093/molbev/msab098 PMC8321534 33822172

[5] Altenhoff, A. M. et al. OMA orthology in 2024: improved prokaryote coverage, ancestral and extant GO enrichment, a revamped synteny viewer and more in the OMA Ecosystem. *Nucleic Acids Res*. 10.1093/nar/gkad1020 (2023). 10.1093/nar/gkad1020 PMC10767875 37962356

[6] Dessimoz, C. et al. OMA, a comprehensive, automated project for the identification of orthologs from complete genome data: introduction and first achievements. In *RECOMB 2005 Workshop on Comparative Genomics* (eds McLysaght, A. & Huson, D. H.) 61–72 (Springer, 2005).

[7] Kirilenko, B. M. et al. Integrating gene annotation with orthology inference at scale. *Science* **380**, eabn3107 (2023). 37104600 10.1126/science.abn3107 PMC10193443

[8] Rossier, V., Vesztrocy, A. W., Robinson-Rechavi, M. & Dessimoz, C. OMAmer: tree-driven and alignment-free protein assignment to subfamilies outperforms closest sequence approaches. *Bioinformatics*10.1093/bioinformatics/btab219 (2021). 10.1093/bioinformatics/btab219 PMC8479680 33787851

[9] Steinegger, M. & Söding, J. Clustering huge protein sequence sets in linear time. *Nat. Commun.* **9**, 2542 (2018). 29959318 10.1038/s41467-018-04964-5 PMC6026198

[10] Altenhoff, A. M. et al. Standardized benchmarking in the quest for orthologs. *Nat. Methods* **13**, 425–430 (2016). 27043882 10.1038/nmeth.3830 PMC4827703

[11] Emms, D. M. & Kelly, S. OrthoFinder: phylogenetic orthology inference for comparative genomics. *Genome Biol.* **20**, 238 (2019). 31727128 10.1186/s13059-019-1832-y PMC6857279

[12] Cosentino, S., Sriswasdi, S. & Iwasaki, W. SonicParanoid2: fast, accurate, and comprehensive orthology inference with machine learning and language models. *Genome Biol.* **25**, 195 (2024). 39054525 10.1186/s13059-024-03298-4 PMC11270883

[13] Schoch, C. L. et al. NCBI Taxonomy: a comprehensive update on curation, resources and tools. *Database* **2020**, baaa062 (2020). 32761142 10.1093/database/baaa062 PMC7408187

[14] Huerta-Cepas, J., Serra, F. & Bork, P. ETE 3: reconstruction, analysis, and visualization of phylogenomic data. *Mol. Biol. Evol.* **33**, 1635–1638 (2016). 26921390 10.1093/molbev/msw046 PMC4868116

[15] Kumar, S. et al. TimeTree 5: an expanded resource for species divergence times. *Mol. Biol. Evol.* **39**, msac174 (2020). 10.1093/molbev/msac174 PMC9400175 35932227

[16] Nevers, Y. et al. Quality assessment of gene repertoire annotations with OMArk. *Nat. Biotechnol.*10.1038/s41587-024-02147-w (2024). 10.1038/s41587-024-02147-w PMC11738984 38383603

[17] Zajac, N. et al. Gene duplication and gain in the trematode *Atriophallophorus winterbourni* contributes to adaptation to parasitism. *Genome Biol* **13**, evab010 (2021). 10.1093/gbe/evab010 PMC7936022 33484570

[18] Katoh, K. & Standley, D. M. MAFFT multiple sequence alignment software version 7: improvements in performance and usability. *Mol. Biol. Evol.* **30**, 772–780 (2013). 23329690 10.1093/molbev/mst010 PMC3603318

[19] Price, M. N., Dehal, P. S. & Arkin, A. P. FastTree 2—approximately maximum-likelihood trees for large alignments. *PLoS ONE* **5**, e9490 (2010). 20224823 10.1371/journal.pone.0009490 PMC2835736

[20] Huerta-Cepas, J., Dopazo, H., Dopazo, J. & Gabaldón, T. The human phylome. *Genome Biol.* **8**, R109 (2007). 17567924 10.1186/gb-2007-8-6-r109 PMC2394744

[21] Vilella, A. J. et al. EnsemblCompara GeneTrees: complete, duplication-aware phylogenetic trees in vertebrates. *Genome Res.* **19**, 327–335 (2009). 19029536 10.1101/gr.073585.107 PMC2652215

[22] Persson, E., Kaduk, M., Forslund, S. K. & Sonnhammer, E. L. L. Domainoid: domain-oriented orthology inference. *BMC Bioinf.* **20**, 523 (2019). 10.1186/s12859-019-3137-2 PMC6816169 31660857

[23] Li, L., Stoeckert, C. J. Jr & Roos, D. S. OrthoMCL: identification of ortholog groups for eukaryotic genomes. *Genome Res.* **13**, 2178–2189 (2003). 12952885 10.1101/gr.1224503 PMC403725

[24] Nevers, Y. et al. OrthoInspector 3.0: open portal for comparative genomics. *Nucleic Acids Res.* **47**, D411–D418 (2019). 30380106 10.1093/nar/gky1068 PMC6323921

[25] Mi, H. et al. PANTHER version 16: a revised family classification, tree-based classification tool, enhancer regions and extensive API. *Nucleic Acids Res.* **49**, D394–D403 (2021). 33290554 10.1093/nar/gkaa1106 PMC7778891

[26] Schreiber, F. & Sonnhammer, E. L. L. Hieranoid: hierarchical orthology inference. *J. Mol. Biol.* **425**, 2072–2081 (2013). 23485417 10.1016/j.jmb.2013.02.018

[27] Altenhoff, A. M. et al. OMA standalone: orthology inference among public and custom genomes and transcriptomes. *Genome Res.* **29**, 1152–1163 (2019). 31235654 10.1101/gr.243212.118 PMC6633268

[28] Altenhoff, A. M., Gil, M., Gonnet, G. H. & Dessimoz, C. Inferring hierarchical orthologous groups from orthologous gene pairs. *PLoS ONE* **8**, e53786 (2013). 23342000 10.1371/journal.pone.0053786 PMC3544860

[29] Train, C.-M., Glover, N. M., Gonnet, G. H., Altenhoff, A. M. & Dessimoz, C. Orthologous Matrix (OMA) algorithm 2.0: more robust to asymmetric evolutionary rates and more scalable hierarchical orthologous group inference. *Bioinformatics* **33**, i75–i82 (2017). 28881964 10.1093/bioinformatics/btx229 PMC5870696

[30] Train, C.-M., Pignatelli, M., Altenhoff, A. & Dessimoz, C. iHam & pyHam: visualizing and processing hierarchical orthologous groups. *Bioinformatics*10.1093/bioinformatics/bty994 (2018). 10.1093/bioinformatics/bty994 PMC6612847 30508066

[31] Nevers, Y. et al. The Quest for Orthologs orthology benchmark service in 2022. *Nucleic Acids Res.* **50**, W623–W632 (2022). 35552456 10.1093/nar/gkac330 PMC9252809

[32] Dylus, D., Altenhoff, A., Majidian, S., Sedlazeck, F. J. & Dessimoz, C. Inference of phylogenetic trees directly from raw sequencing reads using Read2Tree. *Nat. Biotechnol*. 10.1038/s41587-023-01753-4 (2023). 10.1038/s41587-023-01753-4 PMC10791578 37081138

[33] Dylus, D. et al. How to build phylogenetic species trees with OMA. *F1000Res.* **9**, 511 (2020). 35722083 10.12688/f1000research.23790.1 PMC9194518

[34] Altenhoff, A. & Dessimoz, C. Phylogenetic and functional assessment of orthologs inference projects and methods. *PLoS Comput. Biol.* **5**, e1000262 (2009). 19148271 10.1371/journal.pcbi.1000262 PMC2612752

[35] Cosentino, S. & Iwasaki, W. SonicParanoid: fast, accurate and easy orthology inference. *Bioinformatics* **35**, 149–151 (2019). 30032301 10.1093/bioinformatics/bty631 PMC6298048

[36] Zahn-Zabal, M., Dessimoz, C. & Glover, N. M. Identifying orthologs with OMA: a primer. *F1000Res.* **9**, 27 (2020). 32089838 10.12688/f1000research.21508.1 PMC7014581

[37] Emms, D. & Kelly, S. Benchmarking orthogroup inference accuracy: revisiting orthobench. *Genome Biol. Evol.* **12**, 2258–2266 (2020). 33022036 10.1093/gbe/evaa211 PMC7738749

[38] Jones, P. et al. InterProScan 5: genome-scale protein function classification. *Bioinformatics* **30**, 1236–1240 (2014). 24451626 10.1093/bioinformatics/btu031 PMC3998142

[39] Blum, M. et al. The InterPro protein families and domains database: 20 years on. *Nucleic Acids Res.* **49**, D344–D354 (2021). 33156333 10.1093/nar/gkaa977 PMC7778928

[40] Majidian, S. et al. Orthology inference at scale with FastOMA. *Zenodo*10.5281/zenodo.10403053 (2023).

