# Supplementary material for: Orthology inference at scale with FastOMA
Source: Nat Methods. 2025 Jan 3;22(2):269–72. doi: 10.1038/s41592-024-02552-8 (PMC11810774; doi:10.1038/s41592-024-02552-8)
Supplement: Supplementary file 1 — Supplementary Information 1–11, Table 1 and Figs. 1–25. [file 41592_2024_2552_MOESM1_ESM.pdf]

nature methods

Supplementary information

https://doi.org/10.1038/s41592-024-02552-8

Orthology inference at scale with FastOMA

In the format provided by the authors and unedited

Supplementary Information

S1. A note on the definition of HOG
S2. Full benchmarking results for QfO
S3. Comparing FastOMA results with OMAmer mapping
S4. Impact of reference HOG database on FastOMA results
S5. FastOMA robustness on threshold
S6. Impact of OMA database on FastOMA
S7. Impact of species tree on FastOMA
S8. The group benchmarking for the clade Bilateria.
S9. FastOMA's ability to select isoforms
S10. FastOMA's ability to find split genes
S11. FastOMA Nextflow DAG

S1. A note on the definition of HOG

A HOG comprises all the present-day genes that have descended from a single gene in a reference ancestor[35]. Hence, HOGs relate present-day genes in terms of those of ancestral species. For instance, all mammalian insulin genes descended from a single insulin at the root of the mammals. There is thus one insulin HOG at the mammalian level. But within rodents, where insulin is duplicated, the two copies belong to distinct rodent HOGs, nested into the first one (**Supplementary Figure 1**).

HOGs have several conceptual advantages: HOGs provide a precise definition for the useful but vague concepts of gene families and subfamilies. Because each HOG corresponds to an ancestral gene in a given ancestor, they collectively give the gene repertoires of said ancestor. HOGs are a scalable alternative to gene trees, which tend to be hard to infer and interpret.

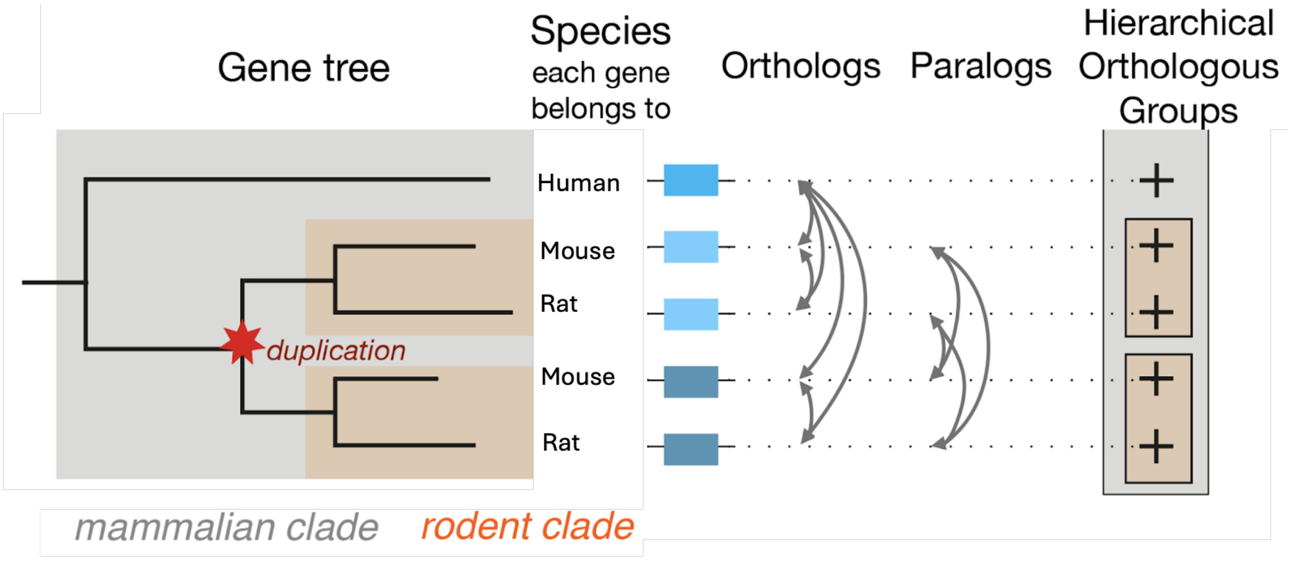

Supplementary Figure 1. An example of HOG structure.

The "rootHOG" corresponds to the deepest ancestral gene and defines a gene family. Of note, rootHOG may correspond to a specific taxonomic level of the species tree (not necessarily the root of the species tree) where it appeared first over the course of evolution. When multiple HOGs exist

in a descendant, they represent distinct gene subfamilies. In a nutshell, HOG is the fundamental underlying evolutionary concept which ties in the concepts of present-day or ancestral genes (HOGs), gene families (rootHOGs), and gene subfamilies (child HOGs).

The standard for reporting HOGs (and most orthology inferences) is the orthoXML format (https://orthoxml.org/) which allows to describe nested orthologs and paralogs groups (**Supplementary Figure 2**). Thus, an orthoXML file is the primary output of FastOMA.

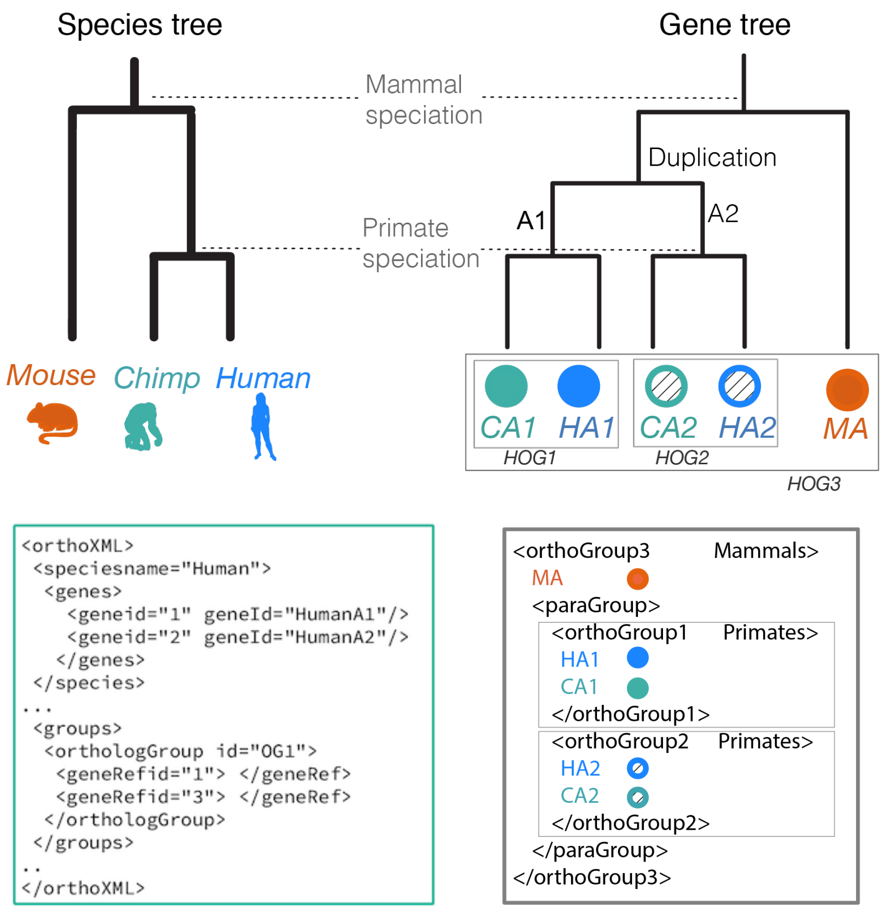

Supplementary Figure 2. An example of HOG structure and orthoXML format.

S2. Full benchmarking results for QfO.

The accuracy of FastOMA was evaluated using the 2020 version of the Quest for Orthologs (QfO) benchmarking dataset[4,31]. This includes 78 species across the tree of life with 984,137 protein sequences for which state-of-the-art orthology inference methods were run. The QfO benchmarks are a series of 11 different tests in three categories including the species discordance test, agreement with reference phylogeny, and functional analysis which are presented below.

S2.1 Species tree discordance test

As one usage of orthology is to infer species trees, as part of the QfO benchmarking, we conducted the species tree discordance test. This evaluates ortholog accuracy by assessing the accuracy of the species tree reconstructed based on it. To decrease the gene-species tree discrepancies due to incomplete lineage sorting, orthologs are sampled from species separated by more than 10 million

years[39]. This test is designed for three clades including Eukaryota, Bacteria and Fungi. The results are provided in **Supplementary Figure 3**.

The subplots a-c in this figure are dedicated to the number of trees that were completed (using orthologous pairs as a proxy for recall) and for which Robinson-Foulds (RF) distance (to compare the topological differences between two trees) were calculated. We use the ETE3 package to calculate this metric. In this benchmark (**Supplementary Figure 3a-c**), FastOMA performs well, with a low average Robinson-Foulds distance (higher precision) and moderate recall, as reflected in the number of completed tree samplings and number of orthologous pairs. This places FastOMA close to the Pareto frontier for three clades of Eukaryota, Fungi, and Bacteria. The subplots **d-f** in **Supplementary Figure 3** show the same benchmark data, but use the fraction of incorrect trees (gene trees with at least one difference from the species tree) as a measure of accuracy and the number of orthologs as a measure of recall.

FastOMA results are similarly close to the Pareto frontier with this measure for Eukaryota and Fungi. It does not perform better than other methods in Bacteria, with the proportion of incorrect trees being higher than other methods with a similar number of orthologs. This might be related to the fact that the FastOMA algorithm for orthology calling assumes vertical descent between genes, an assumption that is not always met in Bacteria. Since the number of species under study in this test is limited, the variance is high. This leads to the generalised species tree discordance test described below.

S2.2 Generalised species tree discordance test

The generalised species tree discordance test (GSTD) includes Eukaryota, Vertebrata, Fungi and LUCA levels. The results reported in **Supplementary Figure 4** are measured in terms of number of orthologous pairs, number of completed tree sampling (as proxies for recall), Robinson-Fold distance, and the fraction of incorrect completed trees (as proxies for precision). In this test, OMA-groups has the highest precision and lowest recall; OrthoMCL and Ensembl Compara are at the other extreme with the highest recall and lowest precision overall. FastOMA consistently has a better recall than other OMA predictions with a higher RF distance compared to OMA-GETHOGs2. Over the benchmark of different clades, it ranks at or close to the Pareto frontier, between the other OMA predictions and most of the other included methods. The relative ranking varies between clades, with FastOMA having slightly lower recall in Vertebrates but higher sensitivity than some OMA predictions for example, but stays true to the general trend overall.

S2.3 Reference gene phylogenies

Another orthology benchmark we exploited is based on the reference gene phylogenies. We used SwissTree, which is a small collection of large- and high-confidence gene family phylogenies with different types of challenges for orthology prediction and species from all domains. In this benchmark, FastOMA performs comparably to other methods, with one of the highest precision (true positive rate: 0.95), but a moderate recall (positive predictive value: 0.69). We also calculated the test for the TreeFam-A reference gene phylogeny which is a larger set of metazoan gene trees covering a taxonomically restricted but wider range of protein families. In this benchmark, FastOMA ranks close to other OMA predictions, with a higher prediction than other tree-based approaches but lower precision and recall than other graph-based predictions. These are reported in **Supplementary Figure 5**.

S2.4 Gene ontology conservation benchmark

The Gene Ontology (GO) conservation benchmark shows how well the Gene Ontology annotations are conserved among the predicted orthologs. This test is based on studies that have demonstrated that orthologs exhibit significant (but moderate) conservation in terms of GO annotation similarity as opposed to paralogs[10]. Therefore, accurate inference of orthology is expected to be associated with gene pairs that are functionally similar at a given evolutionary distance. We assessed functional similarity based on experimentally-backed annotations from the UniProt–Gene Ontology Annotation (GOA) database and Enzyme Commission (EC) numbers from the ENZYME database. To benchmark, we calculated the average Schlicker semantic similarity between GO and EC terms of predicted orthologous pairs as a measure of precision and the number of predicted ortholog relationships as recall[10]. The average Schlicker of FastOMA is 0.465 (0.925) in GO (EC), placing it close to the Pareto frontier.

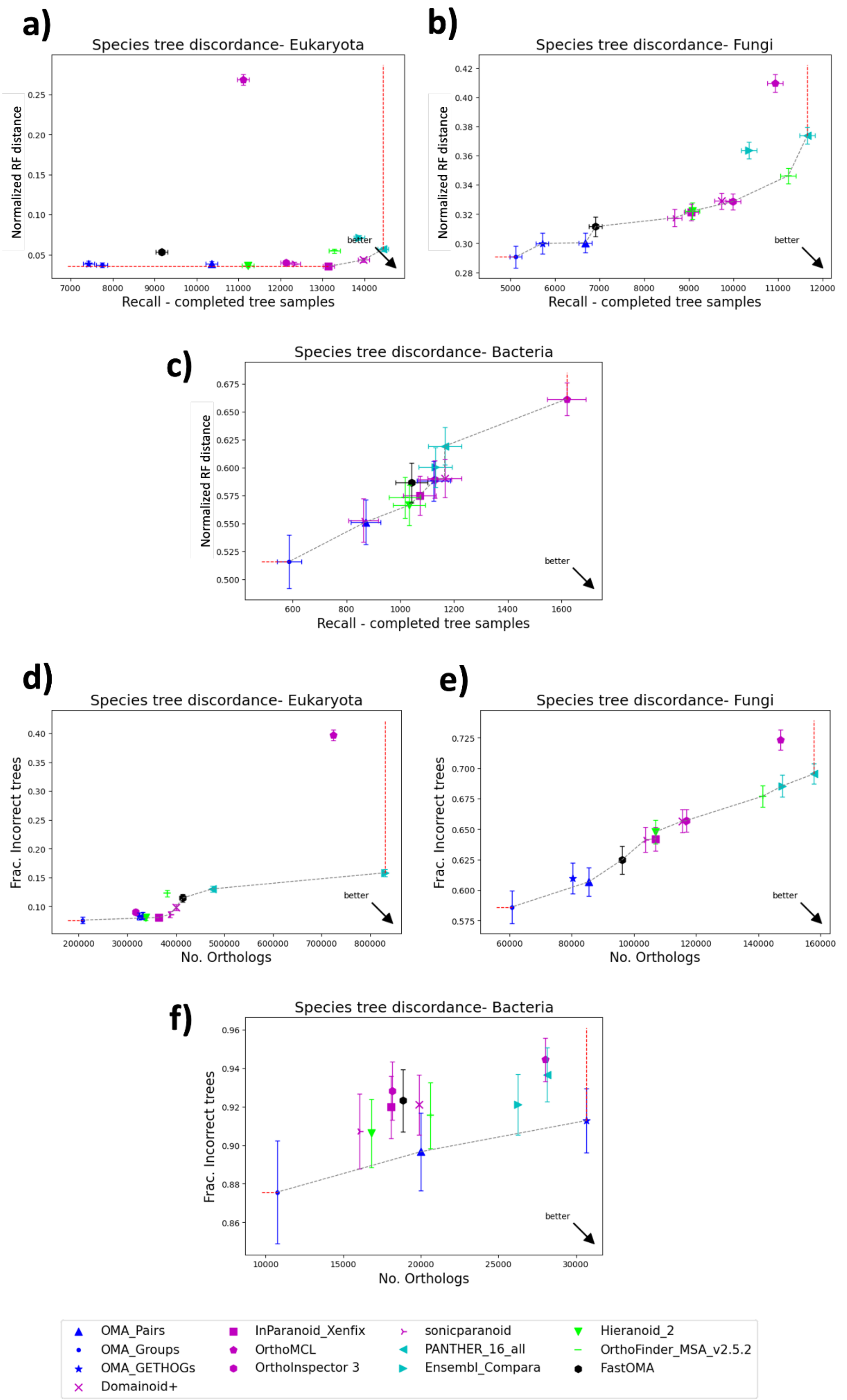

Supplementary Figure 3. The result of the species tree discordance test (a-c) in terms of average Robinson-Foulds distance vs number of completed tree samples (a-c) and in terms of fraction of incorrect trees and number of orthologs (d-f). The other methods of OMA are in blue and the new **FastOMA** is in black. Graph-based methods (OrthoMCL, ORthoInspector, InParanoid, Sonicparanoid, and Domainoid+) are in purple and the tree-based methods are in cyan. The hybrid methods which use both gene tree and graph structure are in green. Number of tree samples for all subplots is 3000. Error bars indicate 95% confidence intervals.

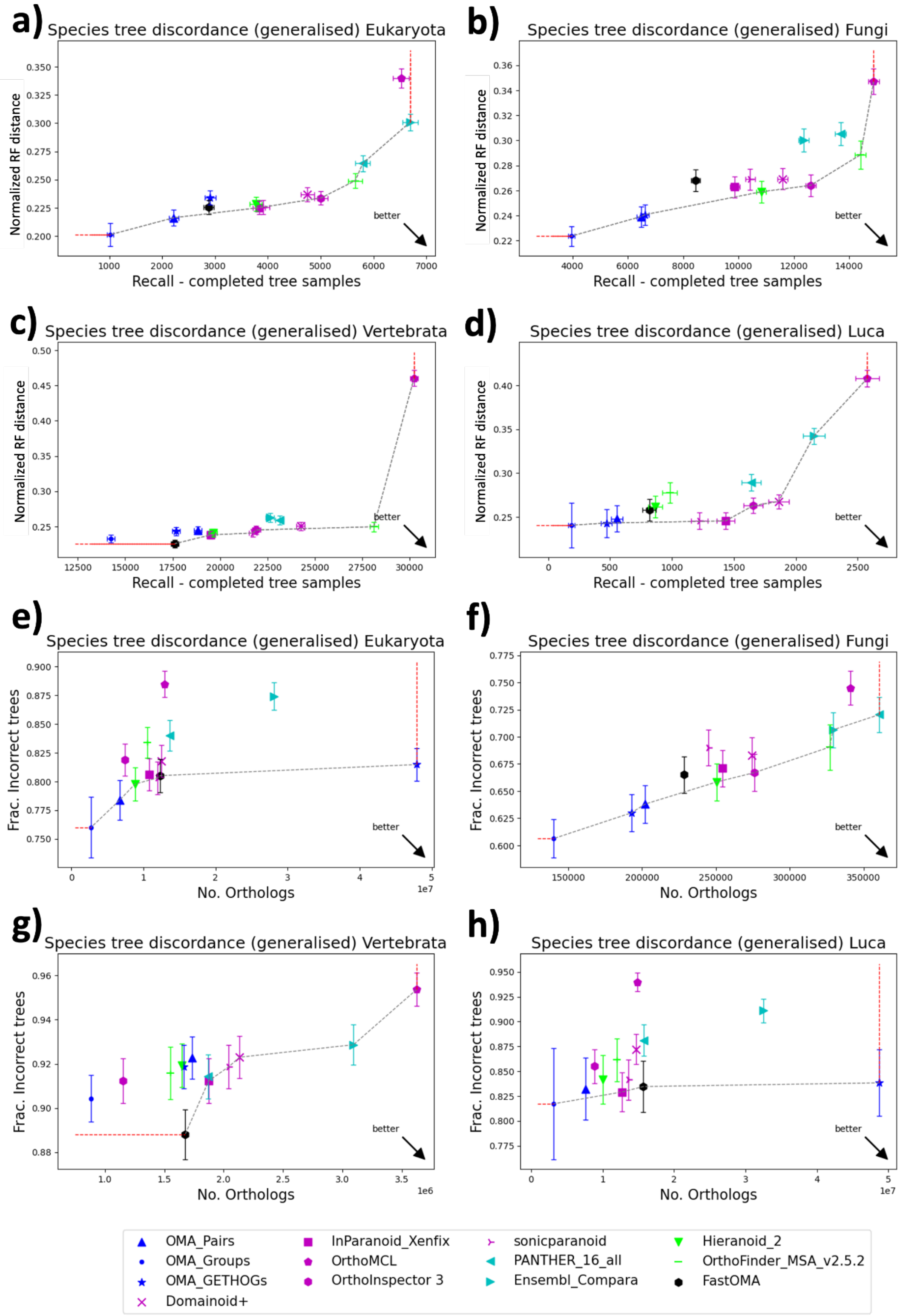

Supplementary Figure 4. The result of generalised species tree discordance test in terms of average Robinson-Foulds distance vs number of completed tree samples n=3000 (a-d) and in terms of the fraction of incorrect trees and number of orthologs (e-h). The other methods of OMA are in blue and the new **FastOMA** is in black. Graph-based methods (OrthoMCL, OrthoInspector, InParanoid, Sonicparanoid, and Domainoid+) are in purple and the tree-based methods are in cyan. The hybrid methods which use both gene tree and graph structure are in green. Error bars indicate 95% confidence intervals.

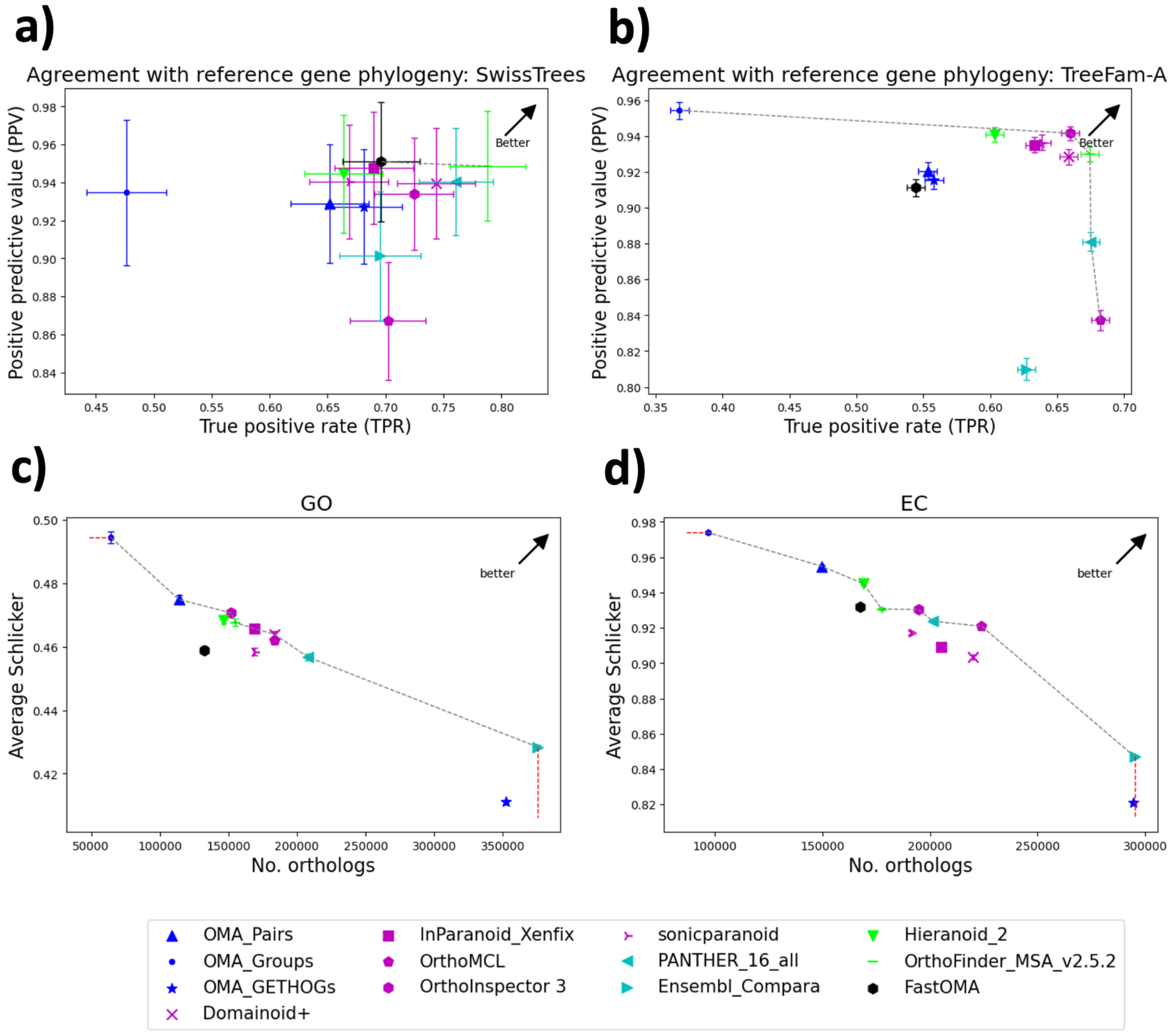

Supplementary Figure 5. The result of agreement with reference gene phylogeny in terms of positive predictive value and true positive rate (a:SwissTree covering 19 trees, b:TreeFam covering 1274 trees. Error bars indicate 95% confidence intervals.). The result of the Functional GO and EC tests in terms of average Schlicker (a similarity score) and number of orthologs (c-d).

S3. Comparing FastOMA results with OMAmer mapping.

FastOMA benefits from OMAmer mapping. However, mapping tools cannot provide enough information for orthology inference. To showcase the superiority of FastOMA against OMAmer mapping, we compared the results of QfO benchmarking tests. Note that the OMAmer tool is solely attributing genes to HOGs (gene family) and thus only predicts homology to other members of the gene family. It cannot differentiate paralogs from orthologs. To find the "orthologous" pairs using OMAmer mapping, we selected the gene with the highest OMAmer score of each species, for each HOG, and we generated orthologous pairs between genes from different species when they are attributed to the same HOG. Gene pairs from the same species are excluded since they are paralogous. We also generated orthologous pairs of genes where one is from a HOG and the other is from its parent HOGs. The results of the QfO species tree discordance benchmarking for such mapping alone show poor performance, with both lower recall and precision than FastOMA and most other orthology methods (**Supplementary Figures 6-8**). This shows the benefit of FastOMA's post-OMAmer-mapping orthology inference algorithm.

S4. Impact of reference HOG database on FastOMA results

Some of the QfO proteomes are already included in the OMAmer database of reference HOGs, which could introduce a bias in FastOMA's favour. To study this effect, we removed such proteomes from the OMAmer database on which we run FastOMA, and used the species discordance benchmark to measure the extent of the bias. Overall, using a database where those proteomes are not present does not significantly affect the results from FastOMA, with most of the difference between versions being within error bars. The QfO results are reported in **Supplementary Figures 6-8**.

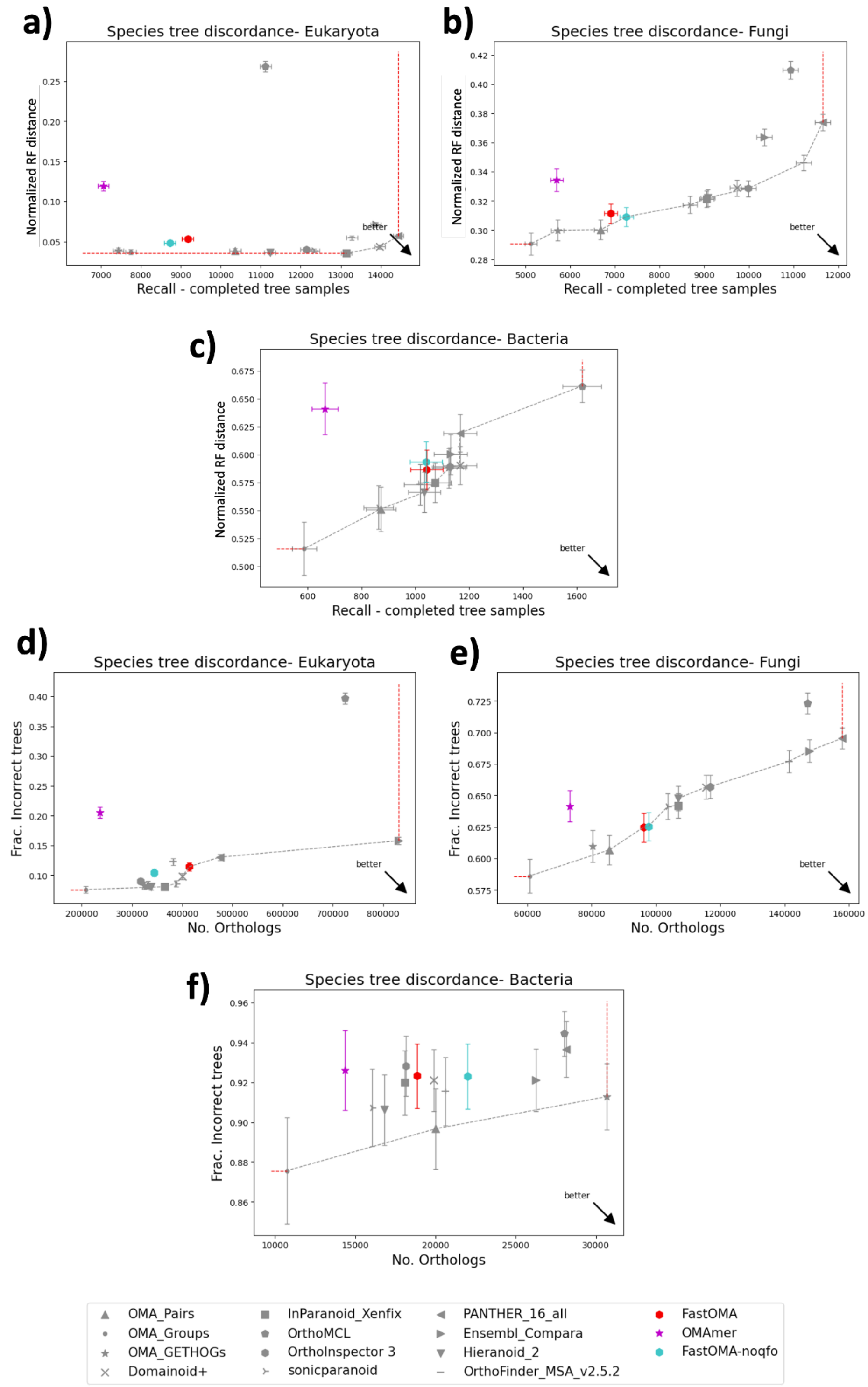

Supplementary Figure 6. The result of the species tree discordance tests comparing OMAmer (pink) and FastOMA including (red)/excluding (cyan) QfO species in the reference set. (a-c): Benchmark results in terms of average Robinson-Foulds distance vs number of completed tree samples n=3000. (d-f): Benchmark results in terms of the fraction of incorrect trees and number of orthologs. Error bars indicate 95% confidence intervals.

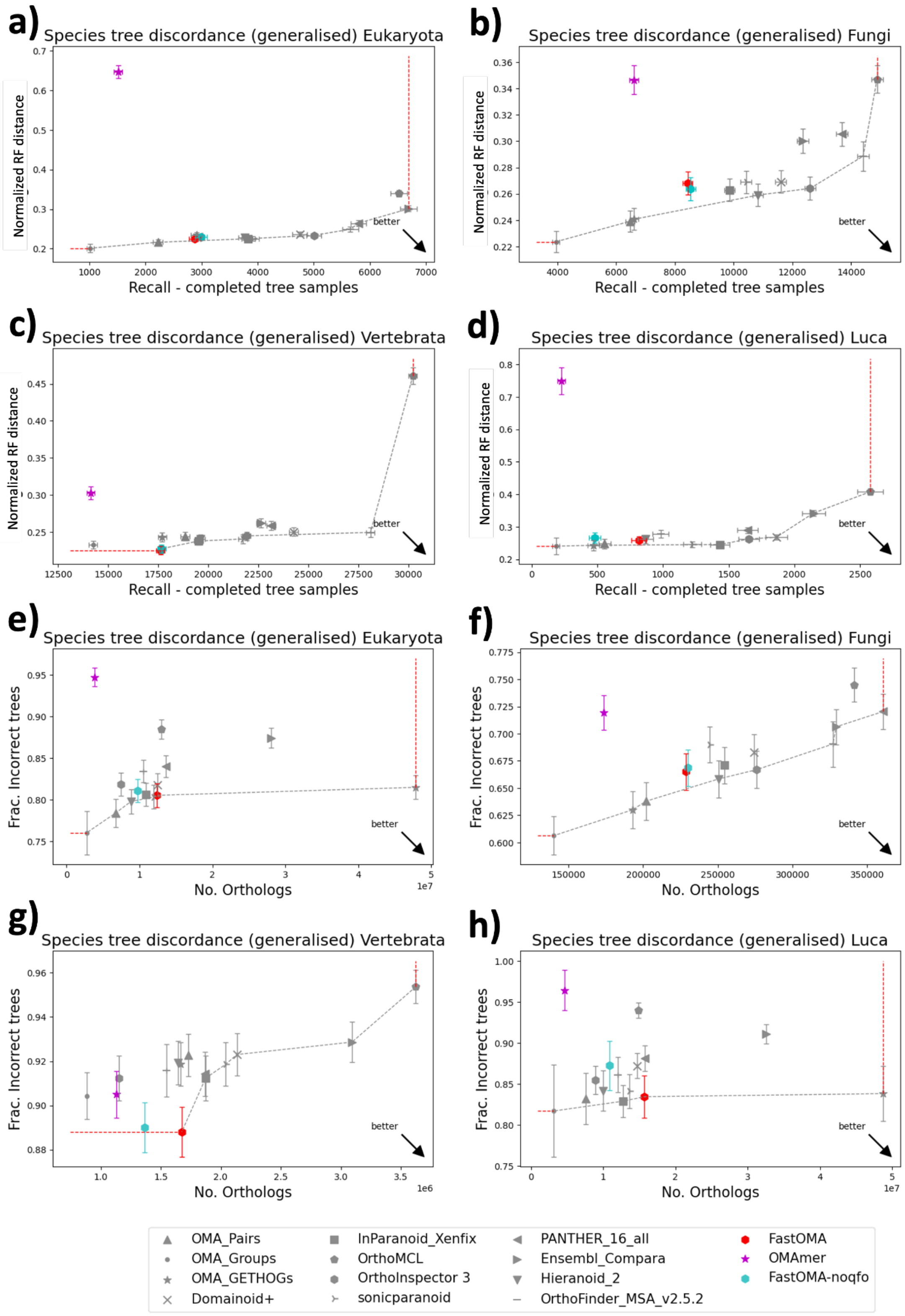

Supplementary Figure 7. The result of the generalised species tree discordance tests comparing OMAmer (pink) and FastOMA including (red)/excluding (cyan) QfO species in the reference set. (a-d): Benchmark results in terms of average Robinson-Foulds distance vs number of completed tree samples n=3000. (e-h): Benchmark results in terms of the fraction of incorrect trees and number of orthologs. Error bars indicate 95% confidence intervals.

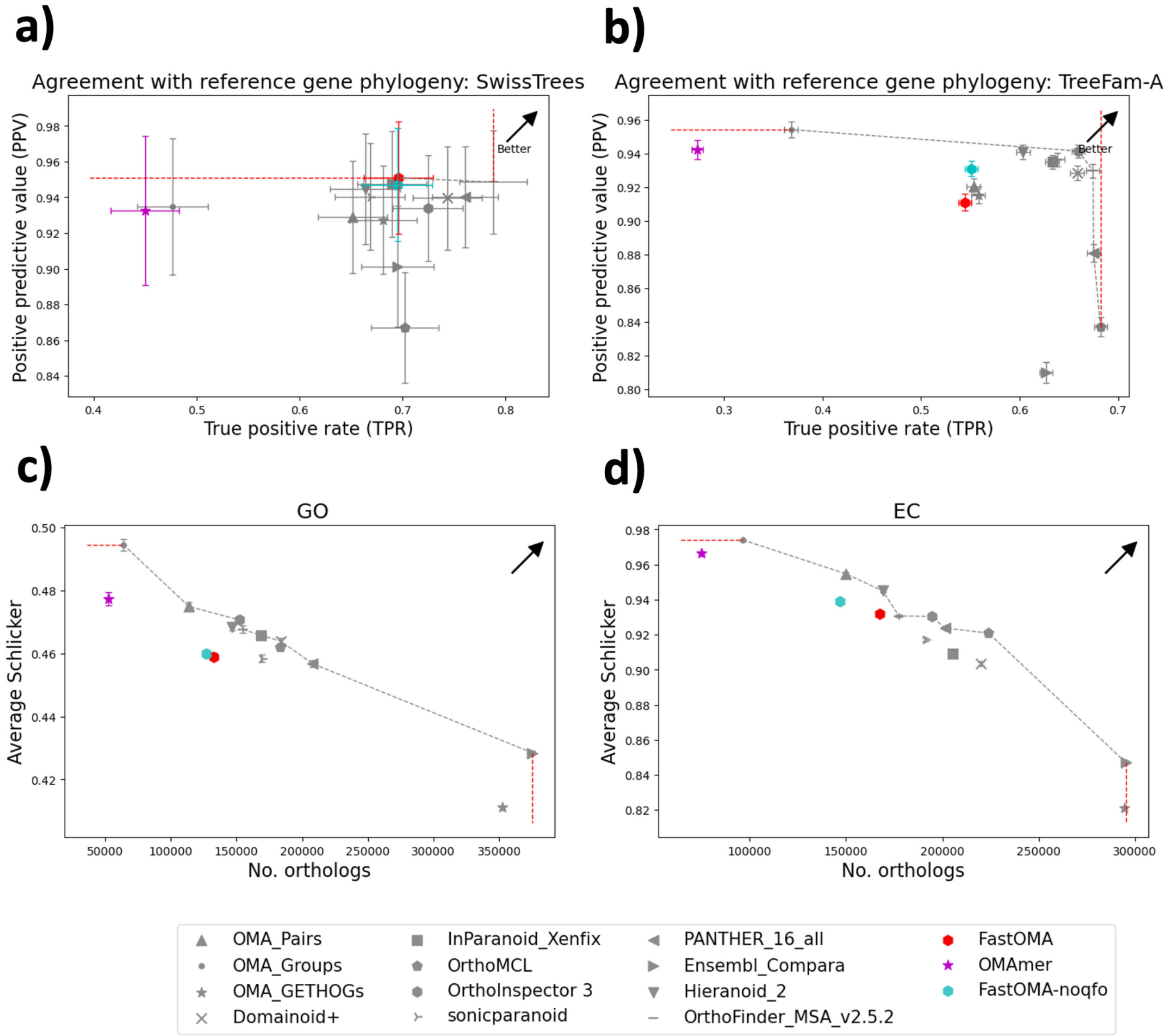

Supplementary Figure 8. The result of the agreement with reference gene phylogeny tree tests (a:SwissTree covering 19 trees, b:TreeFam covering 1274 trees. Error bars indicate 95% confidence intervals.) and functional tests (c-d) comparing OMAmer (pink) and FastOMA including (red)/excluding (cyan) QfO species in the reference set.

S5. FastOMA robustness on threshold

To study the impact of threshold parameters used in FastOMA, we used different parameters and evaluated the results with the QfO species tree discordance tests. Specifically, we changed the thresholds of MSA trimming (row- and column-wise), the score of species overlap method for detecting duplication events and number of proteins sampled per subHOG. Results are reported in **Supplementary Figures 9-11 and 12-17**. The threshold changes only had a minimal effect on the results, with all results being between error bars. FastOMA with default parameters in most cases provide a better tradeoff in terms of precision and recall compared to the other tested parameters.

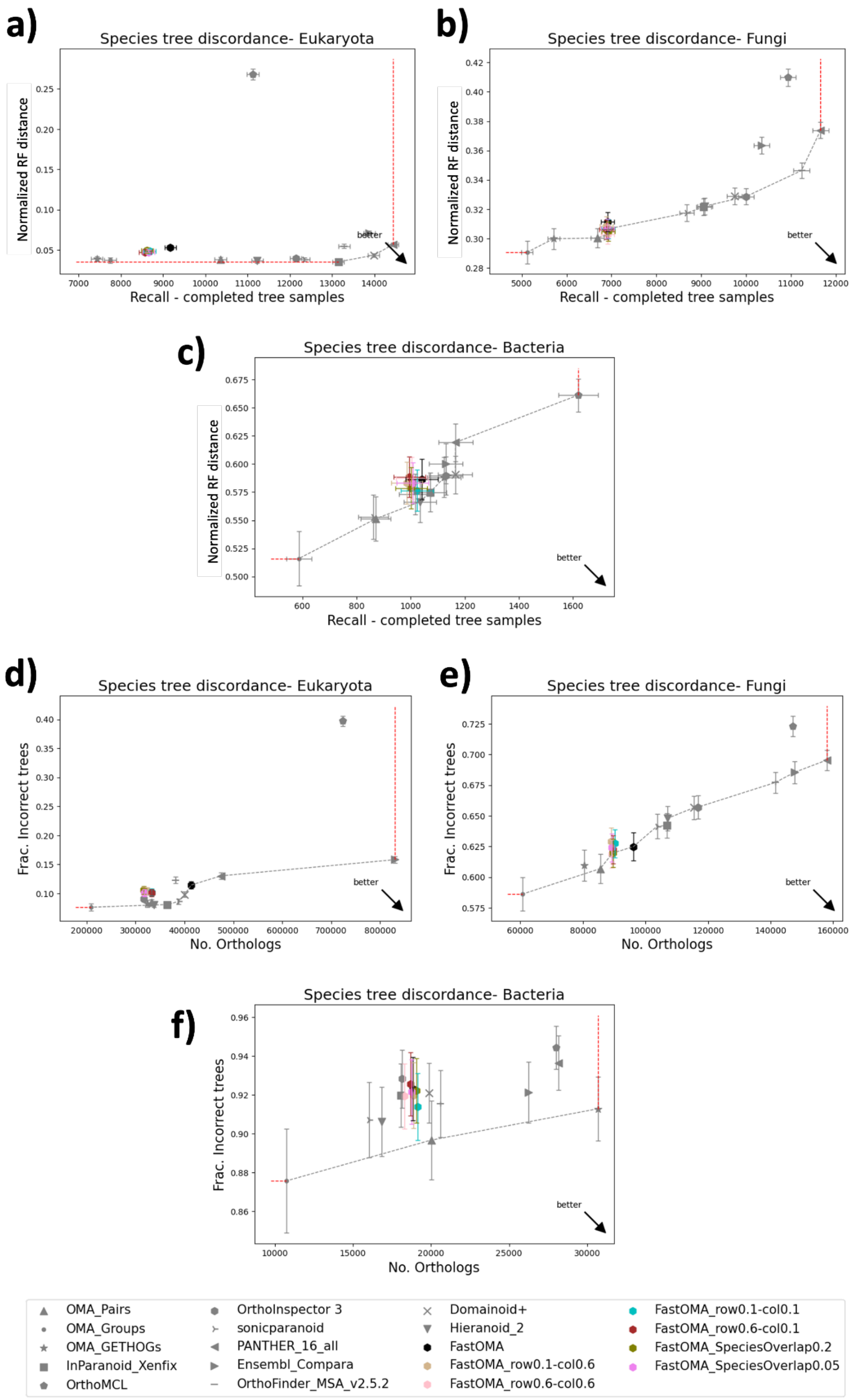

Supplementary Figure 9. The result of species tree discordance tests n=3000, considering different MSA trimming for rows and columns (with threshold of either 0.1 or 0.6) and species overlap scores of 0.2 or 0.05. FastOMA with default values is shown in black; MSA rows trimmed at 0.5, columns trimmed at 0.3, and a species overlap of 0.1. Error bars indicate 95% confidence intervals.

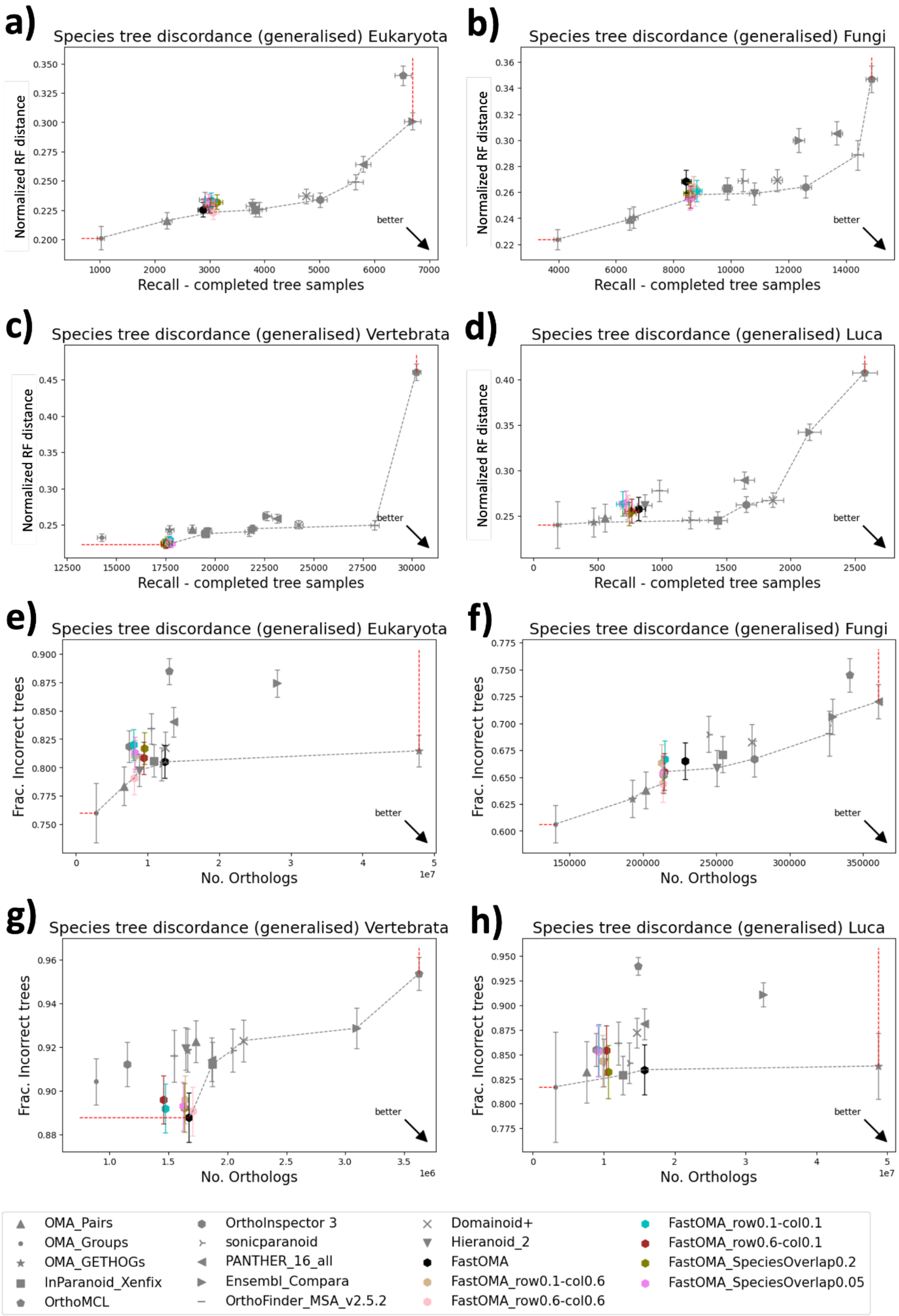

Supplementary Figure 10. The result of generalised species tree discordance tests n=3000 considering different MSA trimming for rows and columns (with threshold of either 0.1 or 0.6) and species overlap scores of 0.2 or 0.05. FastOMA with default values is shown in black; MSA rows trimmed at 0.5, columns trimmed at 0.3, and a species overlap of 0.1. Error bars indicate 95% confidence intervals.

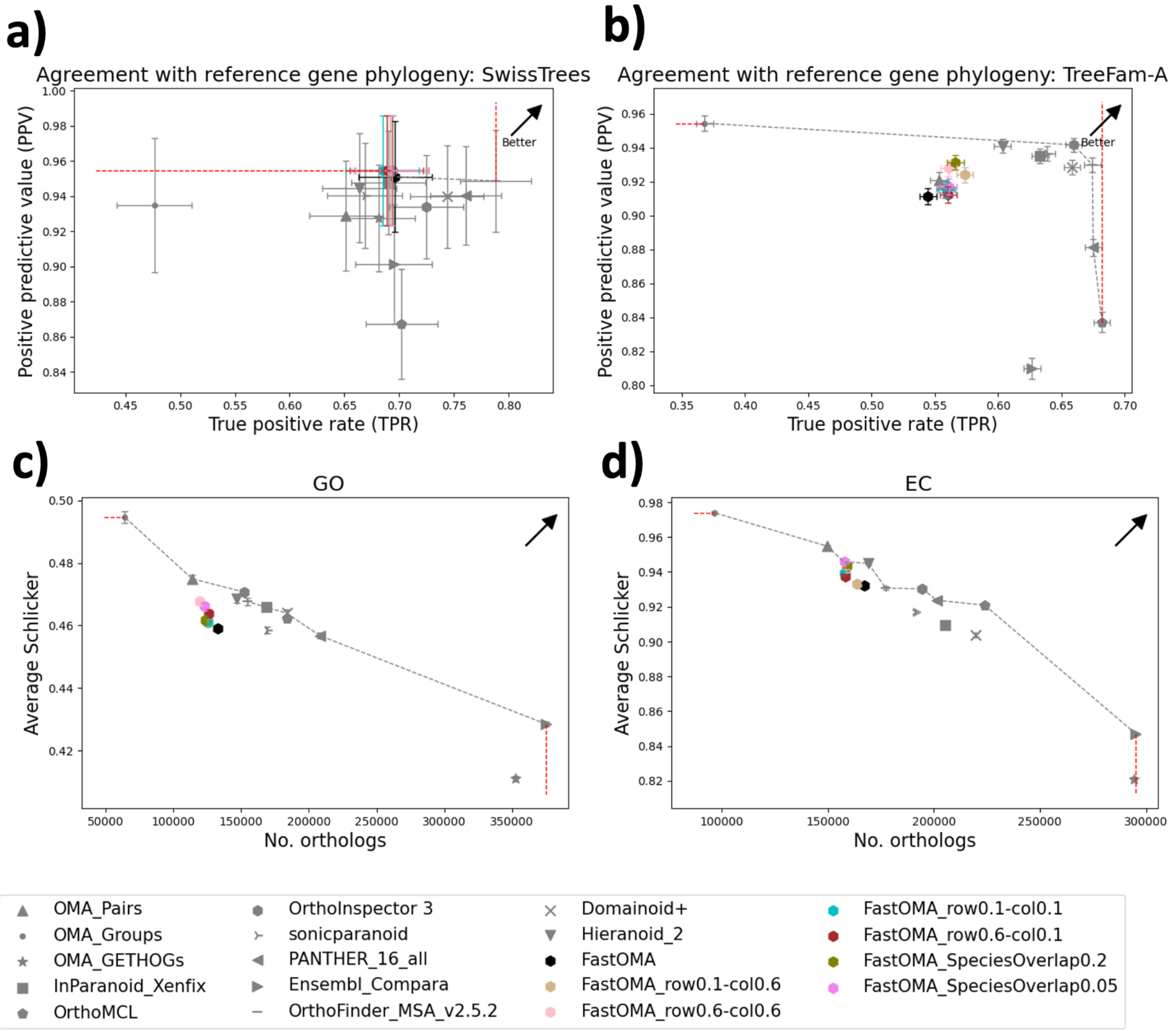

Supplementary Figure 11. The result of agreement with reference gene phylogeny tree tests (a:SwissTree covering 19 trees, b:TreeFam covering 1274 trees. Error bars indicate 95% confidence intervals.) and functional tests (c-d) considering different MSA trimming for rows and columns (with threshold of either 0.1 or 0.6) and species overlap scores of 0.2 or 0.05. FastOMA with default values is shown in black; MSA rows trimmed at 0.5, columns trimmed at 0.3, and a species overlap of 0.1.

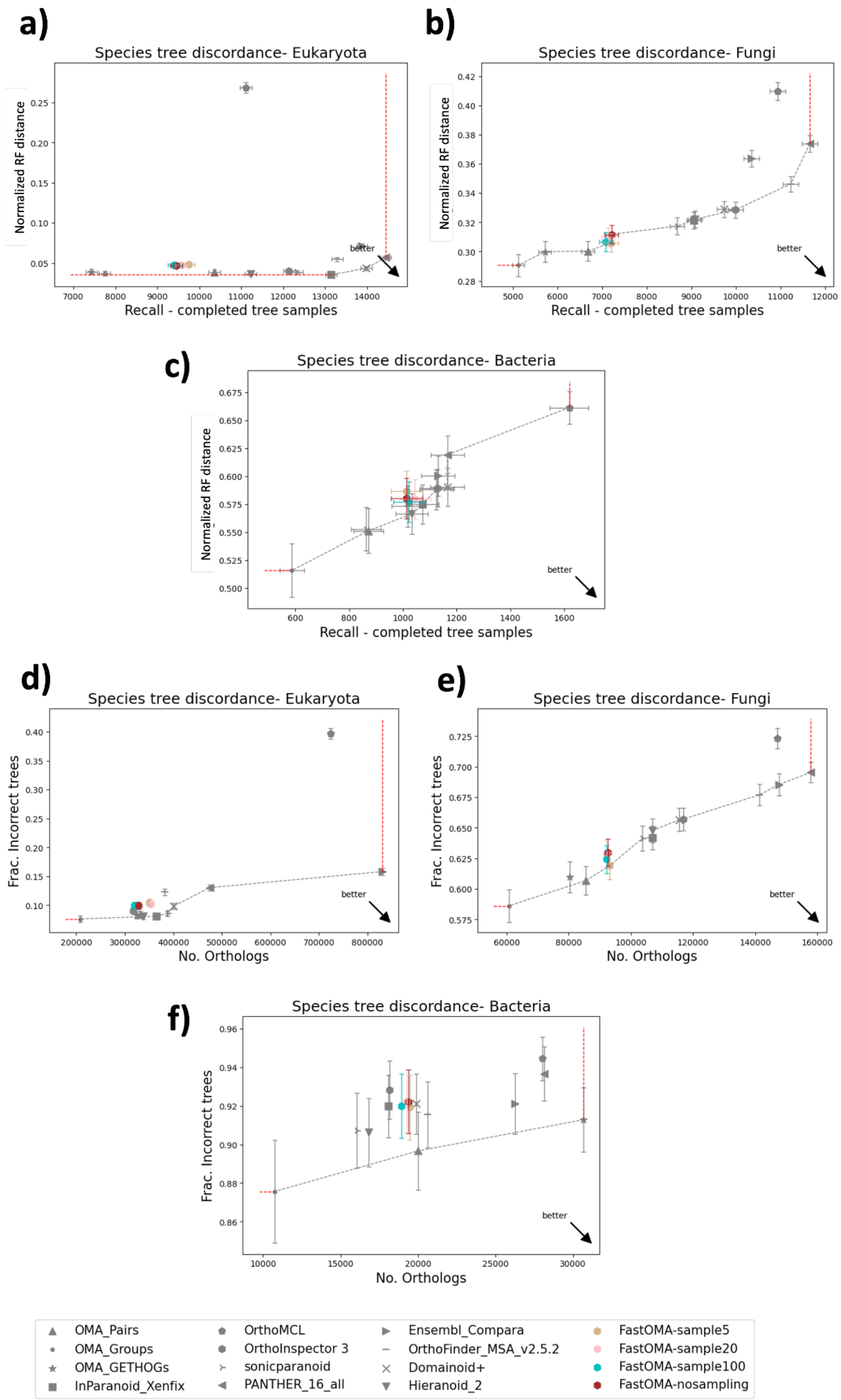

Supplementary Figure 12. The result of species tree discordance tests n=3000, considering different numbers of proteins used in subsampling 5, 20, and 100 and without subsampling. Error bars indicate 95% confidence intervals.

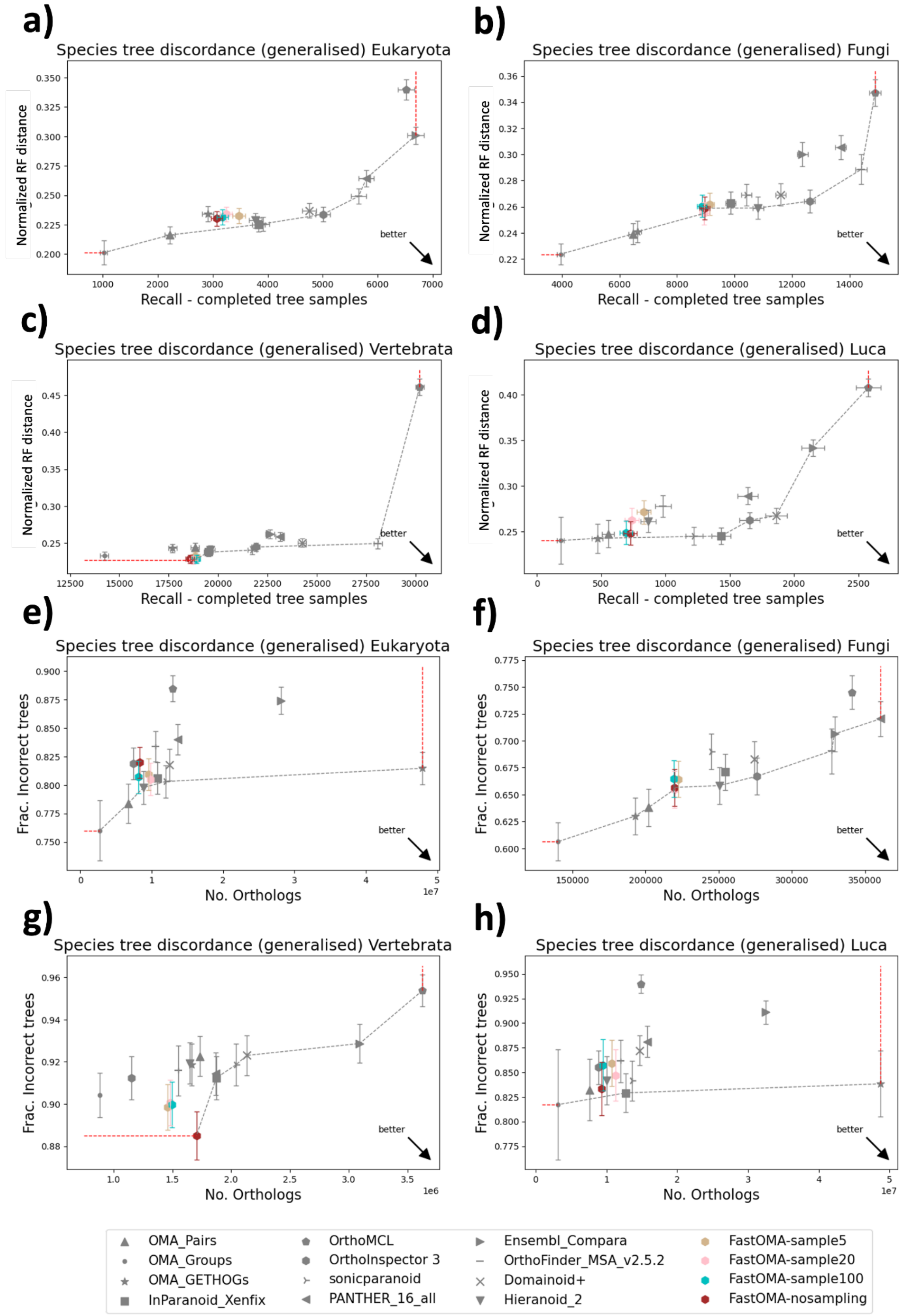

Supplementary Figure 13. The result of generalised species tree discordance tests n=3000, considering different numbers of proteins used in subsampling 5, 20, and 100 and without subsampling. Error bars indicate 95% confidence intervals.

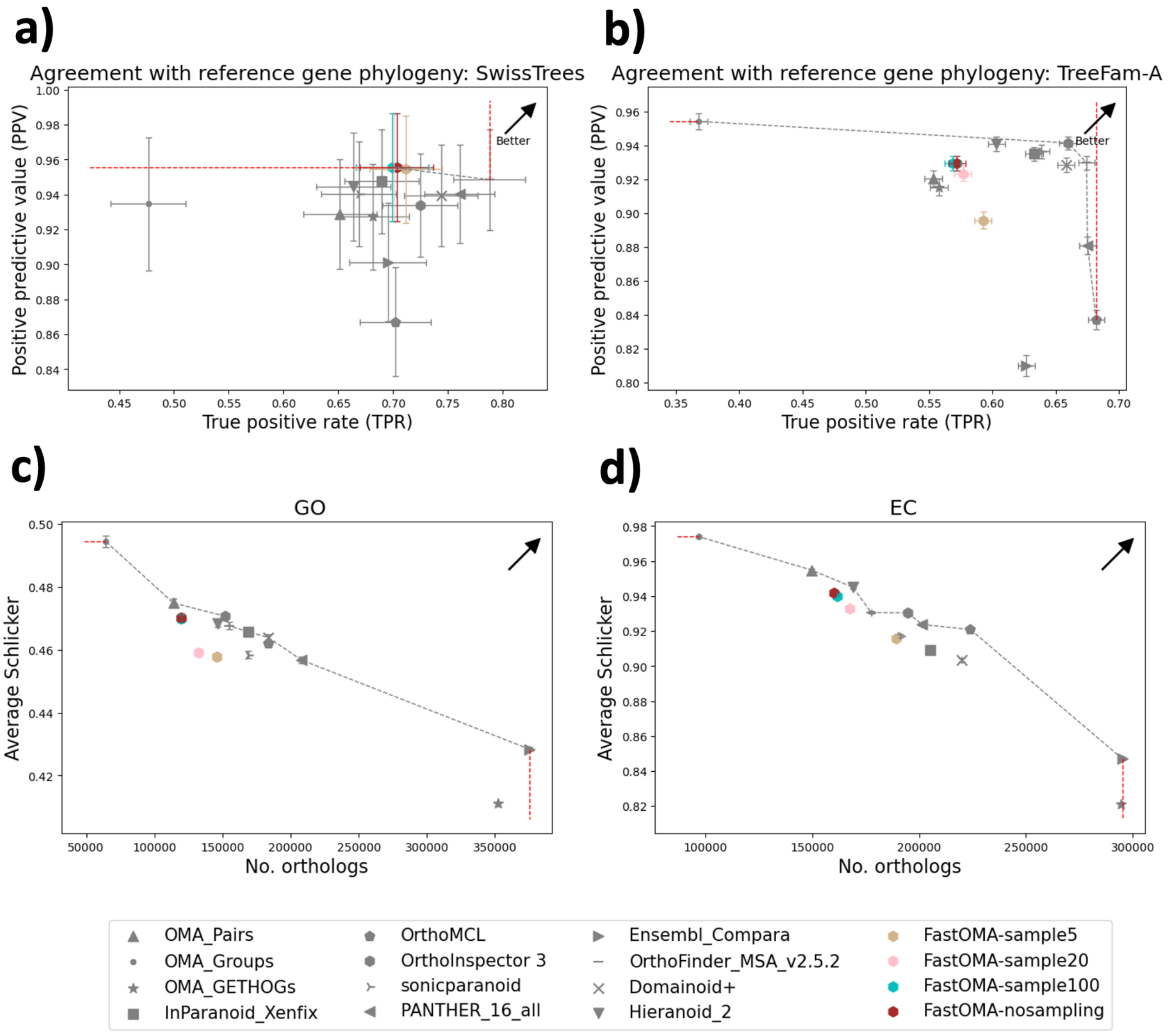

Supplementary Figure 14. The result of agreement with reference gene phylogeny tree tests (a:SwissTree covering 19 trees, b:TreeFam covering 1274 trees. Error bars indicate 95% confidence intervals.) and functional tests (c-d) considering different numbers of proteins used in subsampling 5, 20, and 100 and without subsampling.

S6. Impact of OMA database on FastOMA

In this section, we describe the analysis to investigate how FastOMA is impacted by the reference OMA database. This is implemented by replacing the first step of FastOMA (i.e., OMAmer, which uses the OMA database) with the InterProScan package. The protein grouping (i.e., finding gene families) is a crucial step because if two orthologous genes are mapped to two different groups, there is no chance of rescuing these pairs in subsequent FastOMA steps. This results in false negatives, lowering the recall in orthology inference. Note that FastOMA's grouping is a crucial step for achieving the speed; FastOMA only compares proteins that are inside the gene family, in contrast to other methods that do all-vs-all comparisons.

The benchmarking results provided in Supplementary Figures 15-17 show higher RF distance values and fraction of incorrect trees in most of the discordance tests when comparing FastOMA (OMA database) and FastOMA (InterProScan). However, FastOMA (InterProScan) was able to report more orthologous pairs and better recall. In reference gene families benchmarks, such higher recall was achieved at the expense of a drop in positive predictive values. Over all benchmarks, this strategy leads to overall higher recall but lower accuracy.

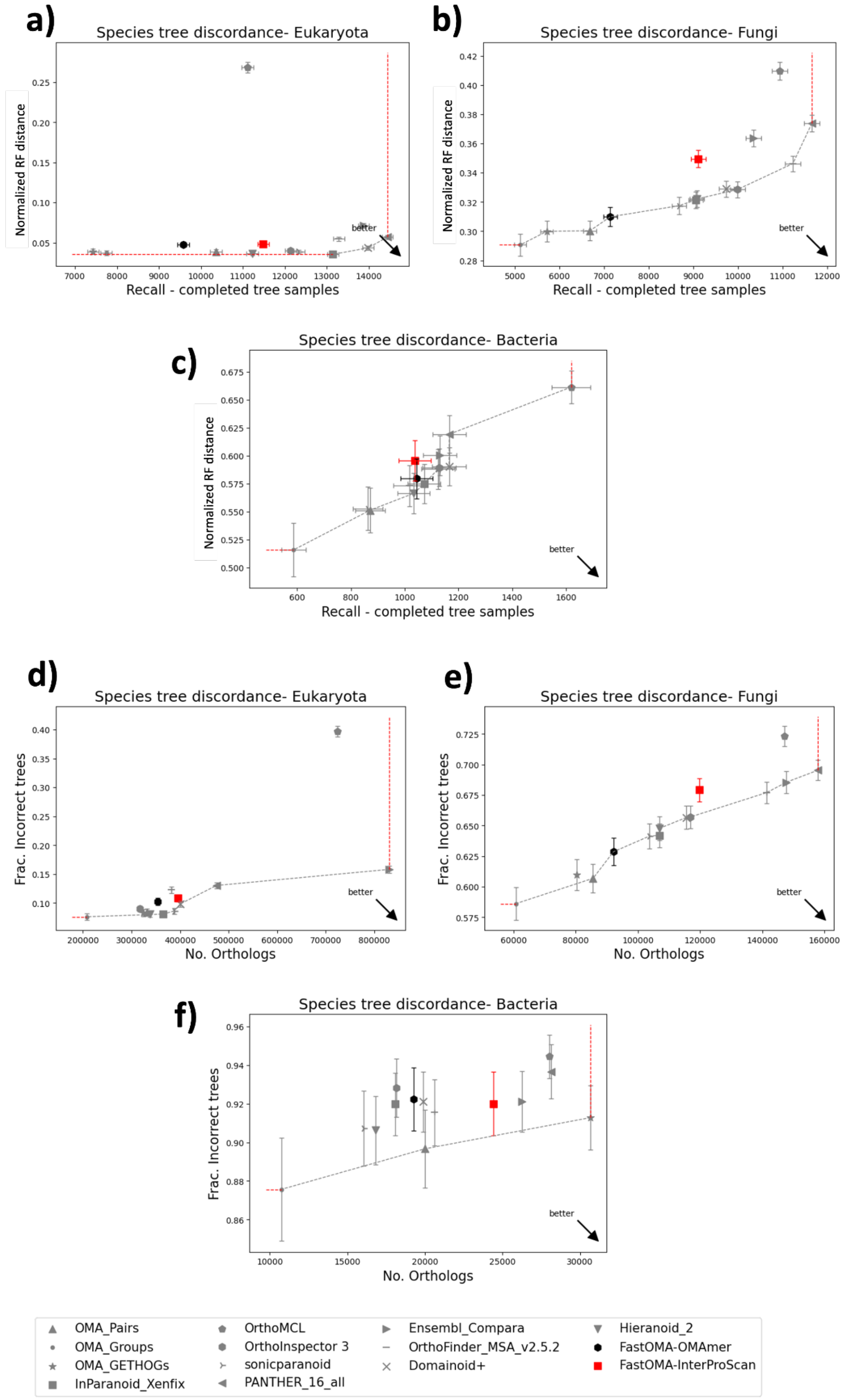

Supplementary Figure 15. The result of species tree discordance tests n=3000, running FastOMA on the InterProScan gene families (without the OMA database). Error bars indicate 95% confidence intervals.

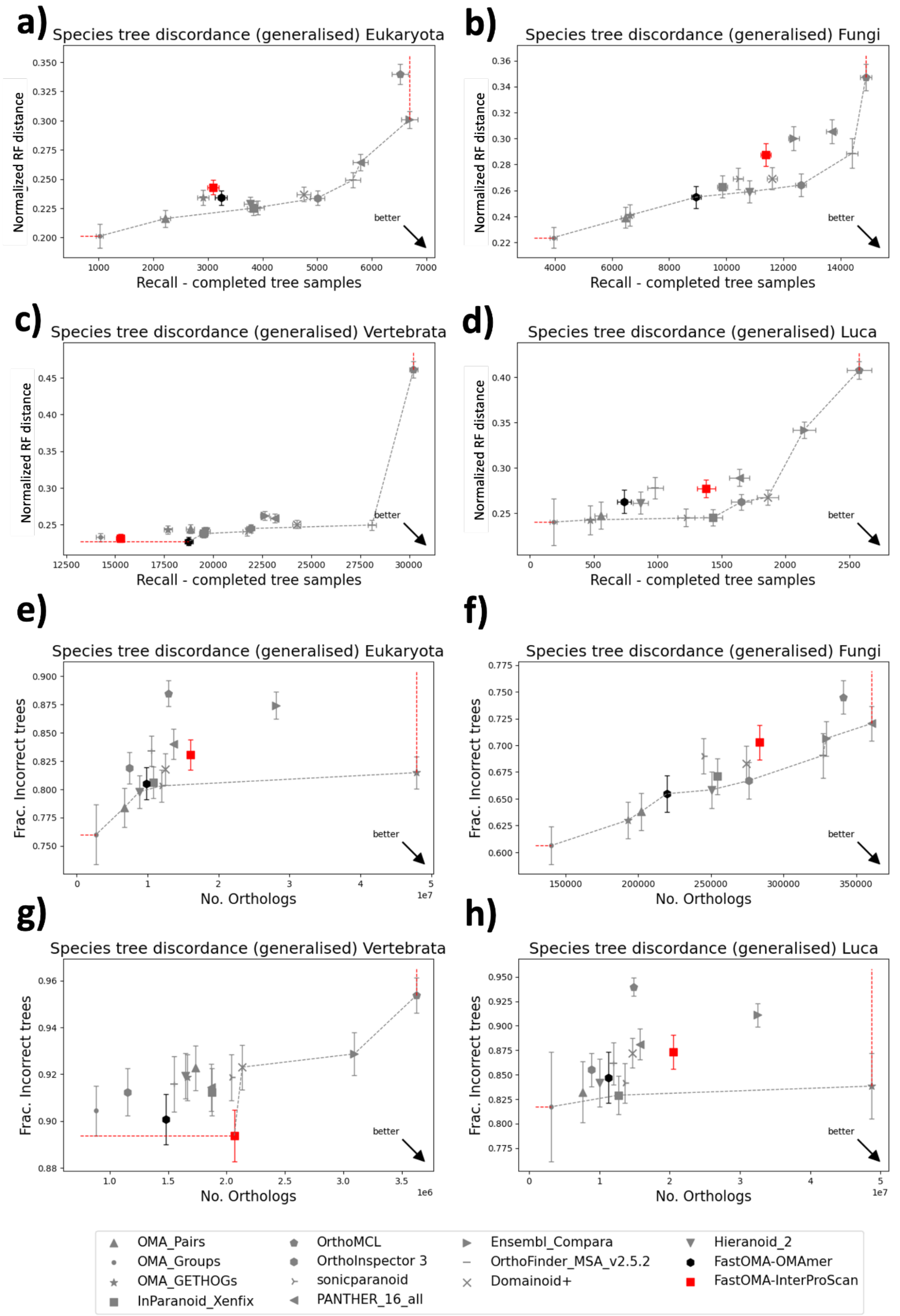

Supplementary Figure 16. The result of generalised species tree discordance tests n=3000, with FastOMA on the InterProScan gene families (without the OMA database). Error bars indicate 95% confidence intervals.

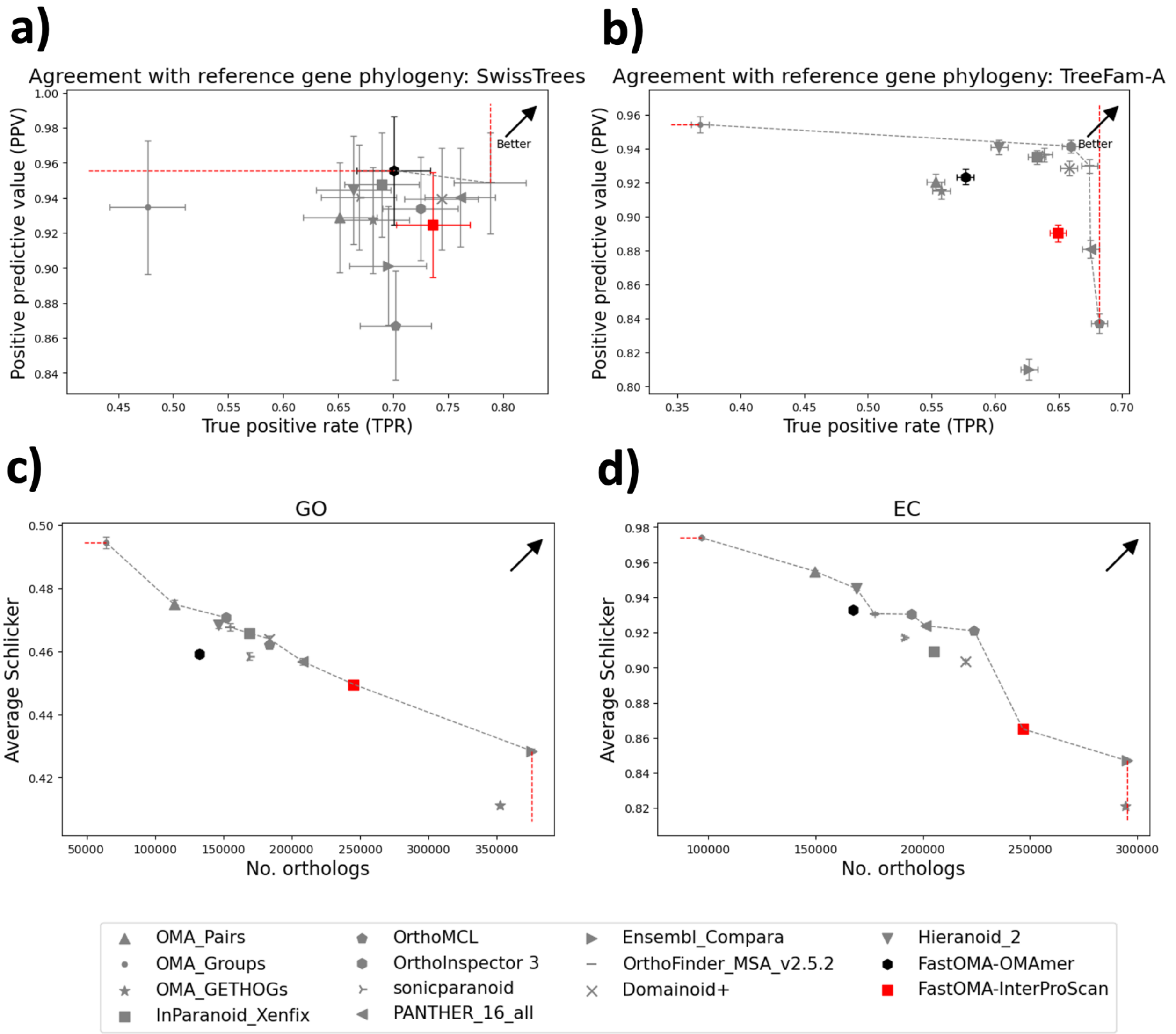

Supplementary Figure 17. The result of agreement with reference gene phylogeny tree tests (a:SwissTree covering 19 trees, b:TreeFam covering 1274 trees. Error bars indicate 95% confidence intervals.) and functional tests (c-d) with FastOMA on the InterProScan gene families (without OMA database).

S7. Impact of species tree on FastOMA

Since one of the FastOMA's inputs is the species tree, we investigated its impact on the orthology inference. As described in the Method section, we swapped five pairs of species (10 species in total) in the QfO dataset. The normalised Robinson-Foulds (RF) distance between the resulting trees and the true QfO species tree were 0.49 and 0.33 for "SwappedTree1" and "SwappedTree2", respectively. This means that nearly half of the branches in SwappedTree1 were spurious. The results of benchmarking tests are shown in Supplementary Figure 18-20. Overall, this major perturbation of the input species tree did result in some performance deterioration, but to a relatively limited extent, and with the tree with more disruption (SwappedTree1) logically resulting in a worse outcome.

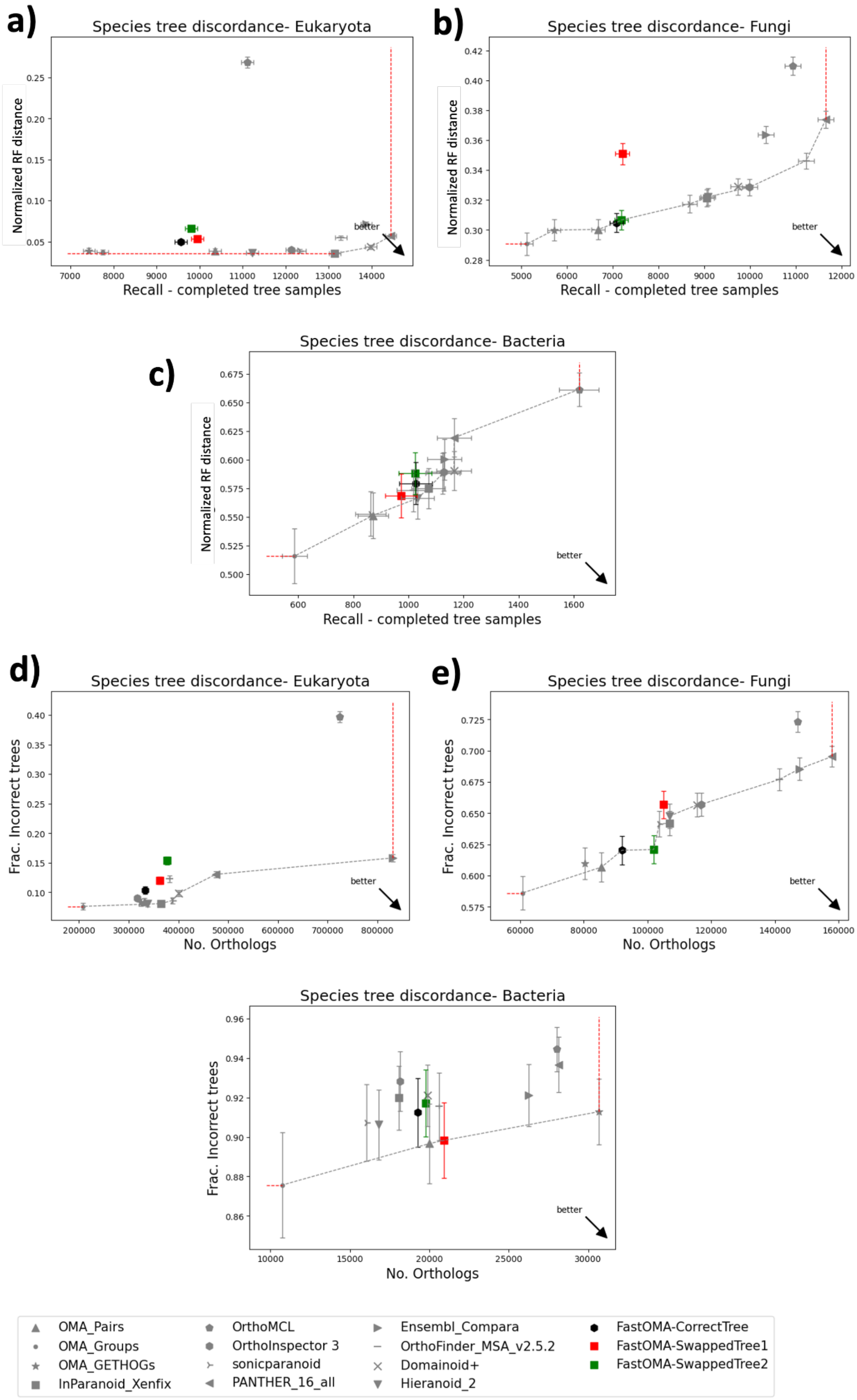

Supplementary Figure 18. The result of species tree discordance tests n=3000, running FastOMA with wrong species trees where five pairs of species are swapped. Error bars indicate 95% confidence intervals.

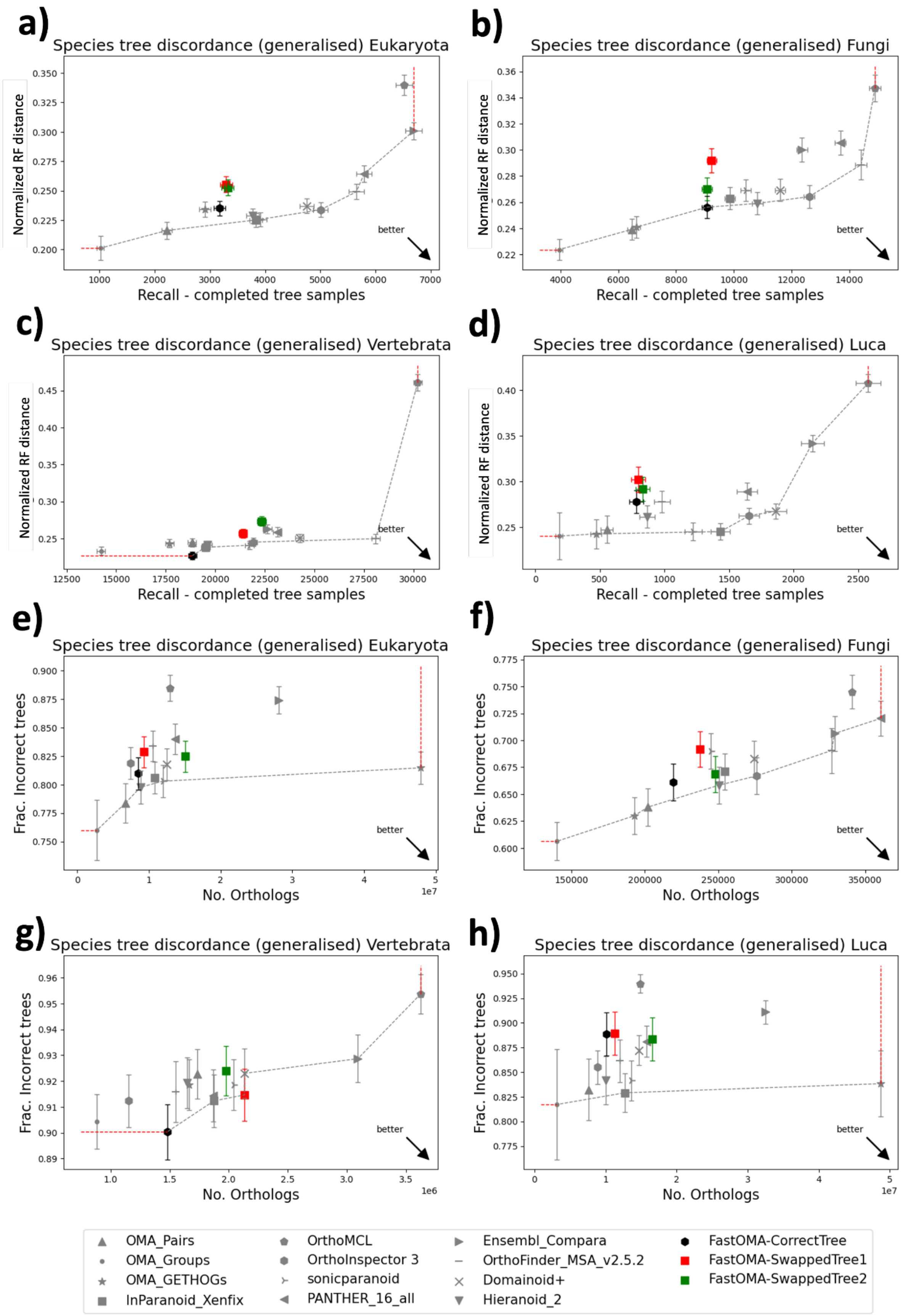

Supplementary Figure 19. The result of generalised species tree discordance tests n=3000, with wrong species trees where five pairs of species are swapped. Error bars indicate 95% confidence intervals.

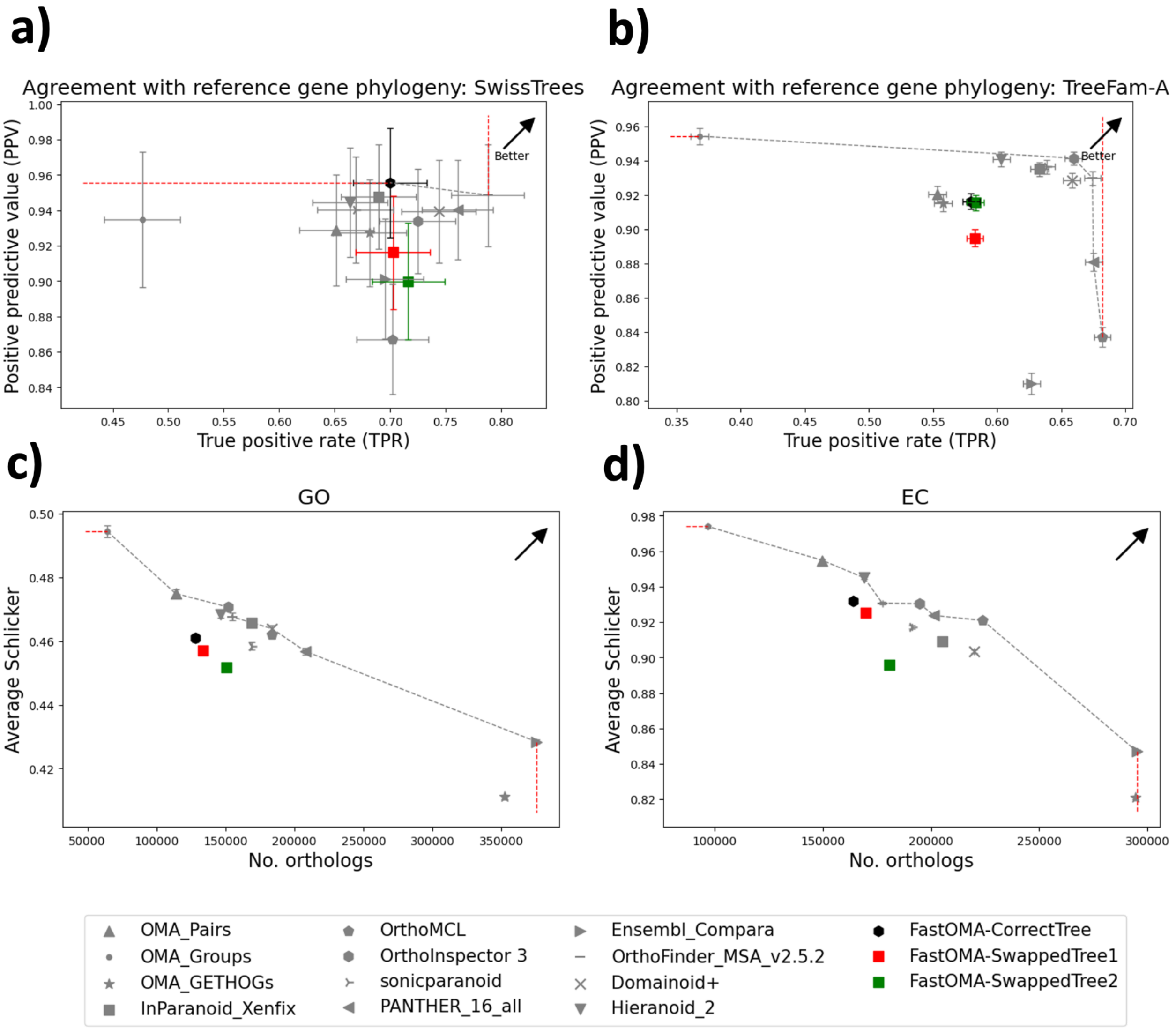

Supplementary Figure 20. The result of agreement with reference gene phylogeny tree tests (a:SwissTree covering 19 trees, b:TreeFam covering 1274 trees. Error bars indicate 95% confidence intervals.) and functional tests (c-d) with wrong species trees where five pairs of species are swapped.

S8. The group benchmarking for the clade Bilateria.

We also used the revisited Orthobench for benchmarking of orthologous groups[40], which has been adapted as part of the QfO benchmarks. This benchmark assesses the ability of orthology inference to accurately predict 70 curated orthologous groups at the Bilateria level. As many of the tools in the QfO benchmark only report the orthology pairs, we could only include Panther and OMA-GETHOGs2 for comparison with FastOMA since these were the only two available as groups of proteins on the QfO public repository. FastOMA has a precision of 0.758 and 0.46 recall; this is a lower precision than OMA-GETHOGs2 with a slightly higher recall, but a higher precision than Panther which has a higher recall.

Supplementary Table 1. The result of group-based benchmarking for the clade Bilateria.

	Panther	OMA-GETHOGs2	FastOMA
PPV (Positive predictive value, precision)	0.58	0.876	0.802
TPR (True positive rate, recall, sensitivity)	0.56	0.43	0.518

S9. FastOMA's ability to select isoforms

In contrast to most other orthology methods, FastOMA considers multiple input alternative splicing isoforms and aims to identify the evolutionarily best-conserved one for orthology inference. FastOMA selects the isoform with the highest OMAmer family score, i.e., the one with the best k-mer similarity with its closest gene family given its length. We compared the results of FastOMA using different ways to select isoforms: choosing the longest one as is often done by other methods, selecting the UniProt reference isoform, and FastOMA's selection. The analysis on UniProt reference proteomes showed that FastOMA's selection resulted in the most parsimonious results, i.e., the least number of rootHOGs and total implied losses (**Supplementary Figure 12**) when reconstructing gene family evolutionary histories. FastOMA selection resulted in the non-longest isoforms being selected for 35% of the proteins with multiple isoforms.

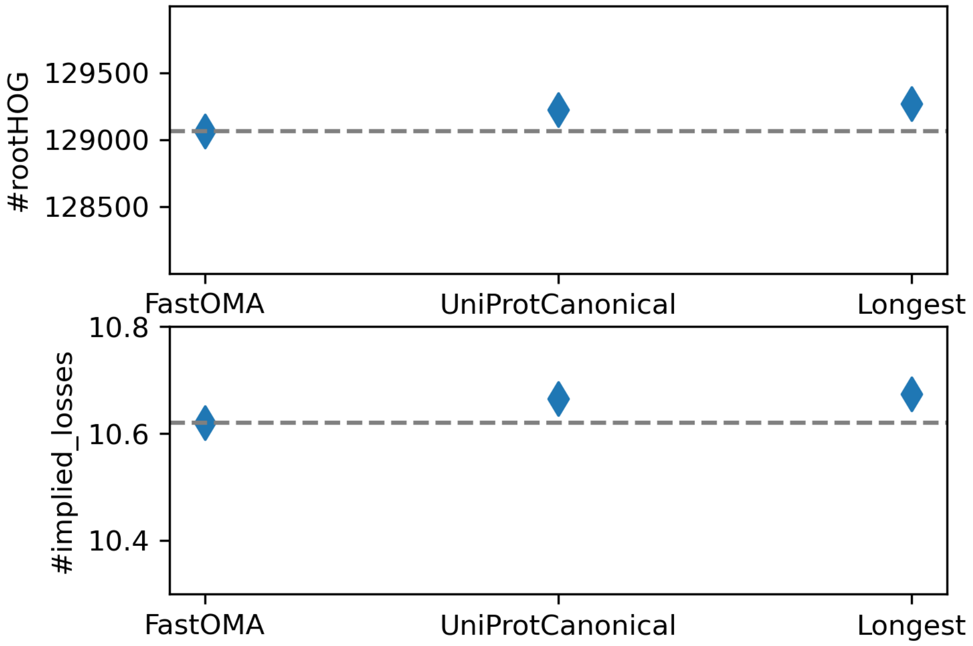

Supplementary Figure 21. Impact of selecting the isoforms on the number of rootHOGs and the total number of implied losses comparing FastOMA's selection with the UniProt Canonical and longest isoforms.

S10. FastOMA's ability to find split genes

FastOMA is capable of finding split genes, i.e., parts of the same gene predicted as multiple different genes, and merging them in the multiple sequence alignment (MSA). This is done to correct issues that might arise due to errors in genome annotation or to fragmented genome assembly where a gene is split across separate scaffolds; two complementary parts of the same gene being included in an MSA would result in an incorrect tree and incorrect labelling of speciation events. Split genes (gene fragments) are found by comparing pairs of genes (rows) in the MSA and those row pairs with complementary gaps and with an overlap in the MSA of less than 15% of alignment length are considered as candidates. These candidate pairs are reported as split genes if they are closer to each other on the gene tree than one fifth of the maximum distance between two leaves of the gene tree, to avoid merging fragments of distant paralogs. They are then merged and considered as a single sequence from the rest of the FastOMA inference, and are reported as such in FastOMA's OrthoXML output. In the UniProt Eukaryote reference proteomes, FastOMA identified 40,297 pairs of sequences (out of 34.4 million sequences) that are likely fragments of split genes, most often found in species with a high proportion of fragments as detected by OMArk and BUSCO. Flagging these split genes aids in cleaning genomic datasets for orthology inference by using more reliable sequences, which in turn will result in a better understanding of genomic architecture and evolution (**Supplementary Figure 22**).

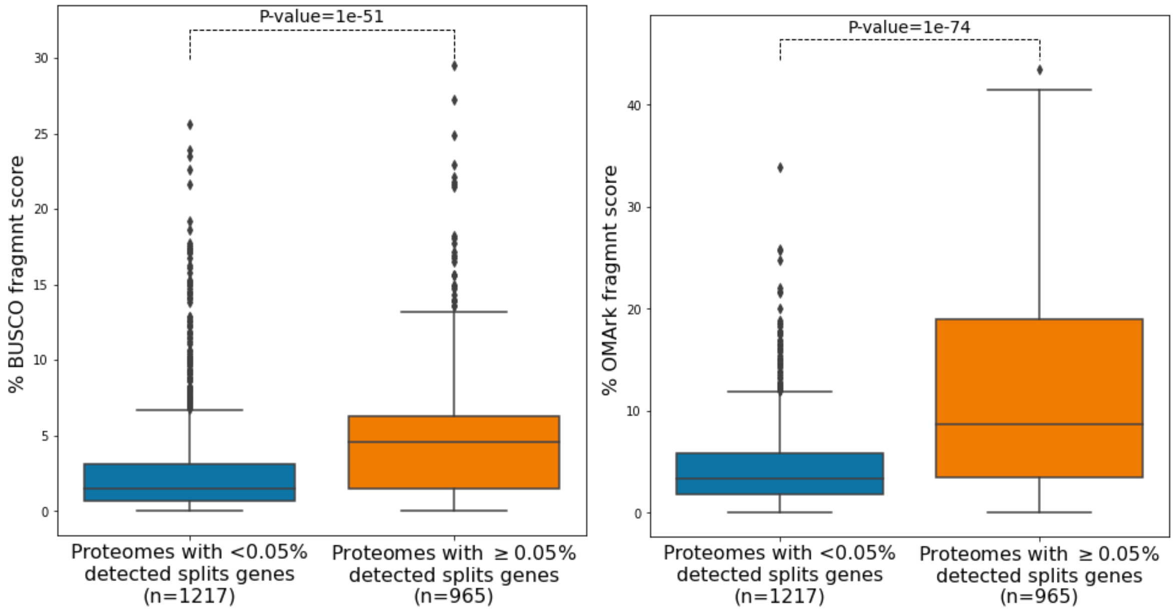

Supplementary Figure 22. FastOMA's ability to find split genes. The y-axis shows the percentage of fragments in proteomes estimated by BUSCO (left) and OMArk (right), partitioned into two groups of proteomes with higher or lower than 0.05% fragments found and merged by FastOMA. The result of the two-sided Mann-Whitney U rank test is reported on top of each figure. Each boxplot represents the distribution where the centre is median, bounds of the box are first and third quartiles and minima and maxima are 1.5x interquartile range.

S11. FastOMA Nextflow DAG

FastOMA benefits from four sub-packages, written in Python. These include *check-input*, *infer-roothogs, batch-roothogs, infer-subhogs,* and *collect-subhogs.* These subpackages are used in a Nextflow pipeline and are dependent on each other through a directed acyclic graph (DAG), visualised below.

As an example, consider a dataset with 10 species and 1004 reference gene families. First, the job *check_input* with one CPU core is submitted. Once finished, 10 *OMAmer* jobs each with one CPU core will be run. *FastOMA.nf* waits until all of them are done. Then, using one CPU core, *infer_roothog* groups the proteins into 1004 families. Next, using one CPU core, *batch_rootHOG* generates (for example) 5 batches of small rootHOGs (each of which includes 200 rootHOGs) and 4 big rootHOGs. Now, *infer_subHOG (*under the wrapper *hog_rest)* starts for the 5 batches (each with one CPU core) and 4 big rootHOGs (under the wrapper *hog_big,* each with 6 CPU cores) totaling 5+24=29 CPU cores. Once all batches are finished, *collect_suhog* reads the output of each batch and writes the final OrthoXML file.

If the total number of needed CPU cores exceeds 500, Nextflow submits the first 500 jobs and sequentially adds one more job, once a job finishes. Start of a job relies on the availability of computation nodes assigned with Slurm Workload Manager on a High Performance Computing cluster. When a job finishes with an error due to time/memory limits, FastOMA re-submits it by doubling the CPU, time or memory. FastOMA also tries to decide the number of CPU cores and memory based on the size of the gene families in the batch. The number of CPUs per task can be changed with the Nextflow configuration file available at our GitHub *FastOMA/conf/base.config*.

In short, FastOMA tries to parallelise the steps as much as possible (with the goal of optimising the wall-clock time) considering the dependencies, using a different number of CPU cores for each step, specified based on the number of species and gene families.

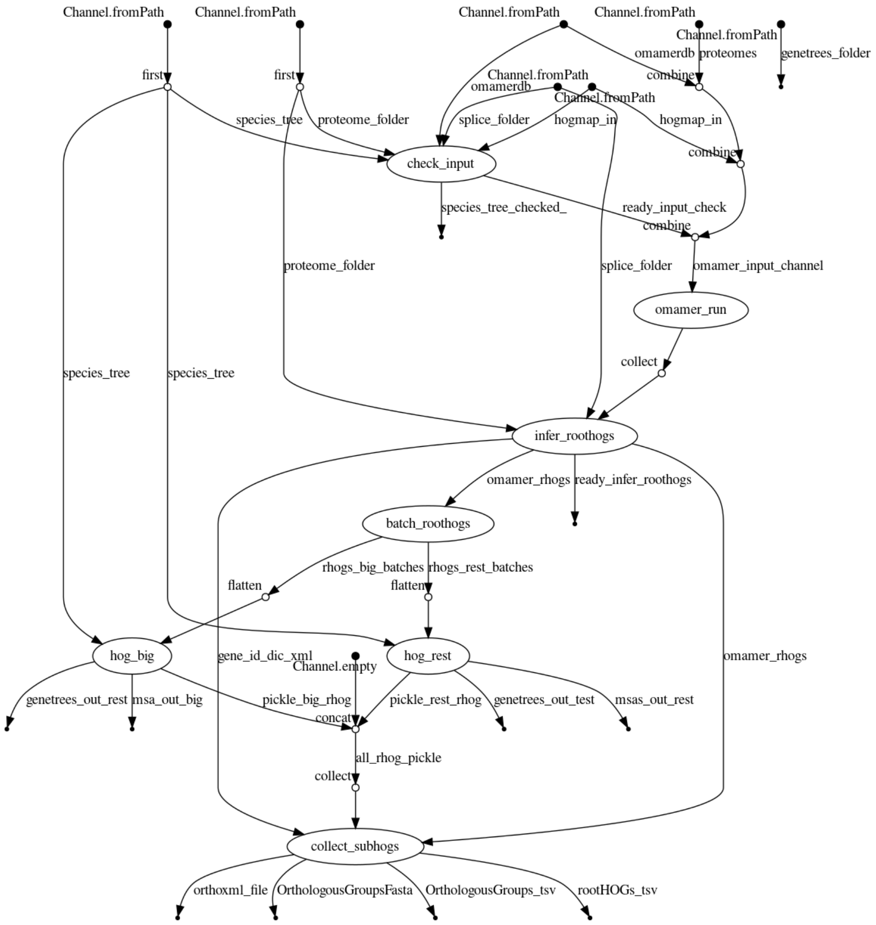

Supplementary Figure 23. Subpackages of the FastOMA software.

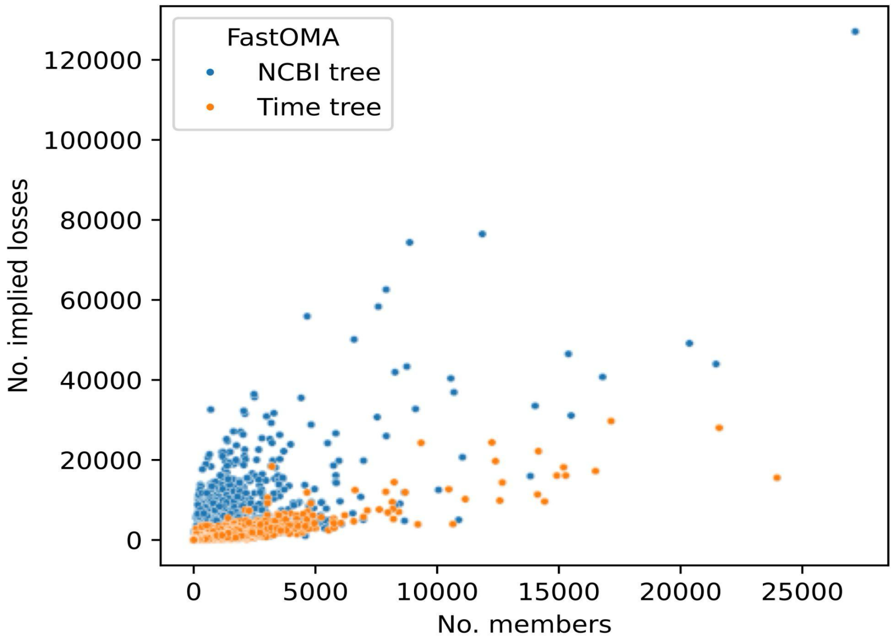

Supplementary Figure 24. The impact of species tree resolution on evolutionary events in terms of implied losses. This is the full data of the figure shown in Figure 2d. Each point corresponds to a rootHOG. Number of rootHOGs that FastOMA found using the NCBI tree is 39,4516 and 38,5697 with the TimeTree.

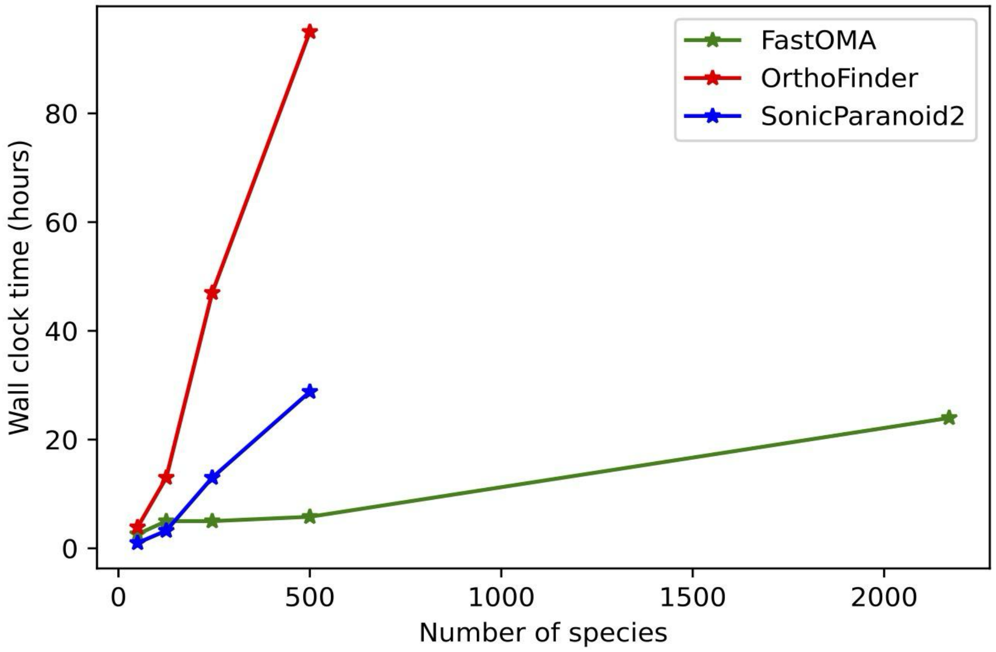

Supplementary Figure 25. The comparison of tools in terms of Wall-clock time in hours.

39. Altenhoff, A. M. & Dessimoz, C. Phylogenetic and functional assessment of orthologs inference projects and methods. *PLoS Comput. Biol.* **5**, e1000262 (2009).
40. Emms, D. M. & Kelly, S. Benchmarking Orthogroup Inference Accuracy: Revisiting Orthobench. *Genome Biol. Evol.* **12**, 2258–2266 (2020).
